# Using text mining for study identification in systematic reviews: a systematic review of current approaches

**DOI:** 10.1186/2046-4053-4-5

**Published:** 2015-01-14

**Authors:** Alison O’Mara-Eves, James Thomas, John McNaught, Makoto Miwa, Sophia Ananiadou

**Affiliations:** Evidence for Policy and Practice Information and Coordinating (EPPI)-Centre, Social Science Research Unit, UCL Institute of Education, University of London, London, UK; The National Centre for Text Mining and School of Computer Science, Manchester Institute of Biotechnology, University of Manchester, 131 Princess Street, Manchester, M1 7DN UK; Toyota Technological Institute, 2-12-1 Hisakata, Tempaku-ku, Nagoya, 468-8511 Japan

**Keywords:** Text mining, Automation, Screening, Study selection, Review efficiency

## Abstract

**Background:**

The large and growing number of published studies, and their increasing rate of publication, makes the task of identifying relevant studies in an unbiased way for inclusion in systematic reviews both complex and time consuming. Text mining has been offered as a potential solution: through automating some of the screening process, reviewer time can be saved. The evidence base around the use of text mining for screening has not yet been pulled together systematically; this systematic review fills that research gap. Focusing mainly on non-technical issues, the review aims to increase awareness of the potential of these technologies and promote further collaborative research between the computer science and systematic review communities.

**Methods:**

Five research questions led our review: what is the state of the evidence base; how has workload reduction been evaluated; what are the purposes of semi-automation and how effective are they; how have key contextual problems of applying text mining to the systematic review field been addressed; and what challenges to implementation have emerged?

We answered these questions using standard systematic review methods: systematic and exhaustive searching, quality-assured data extraction and a narrative synthesis to synthesise findings.

**Results:**

The evidence base is active and diverse; there is almost no replication between studies or collaboration between research teams and, whilst it is difficult to establish any overall conclusions about best approaches, it is clear that efficiencies and reductions in workload are potentially achievable.

On the whole, most suggested that a saving in workload of between 30% and 70% might be possible, though sometimes the saving in workload is accompanied by the loss of 5% of relevant studies (i.e. a 95% recall).

**Conclusions:**

Using text mining to prioritise the order in which items are screened should be considered safe and ready for use in ‘live’ reviews. The use of text mining as a ‘second screener’ may also be used cautiously. The use of text mining to eliminate studies automatically should be considered promising, but not yet fully proven. In highly technical/clinical areas, it may be used with a high degree of confidence; but more developmental and evaluative work is needed in other disciplines.

**Electronic supplementary material:**

The online version of this article (doi:10.1186/2046-4053-4-5) contains supplementary material, which is available to authorized users.

## Background

### The problem: lack of precision in systematic searches

Systematic reviews are a widely used method to bring together the findings from multiple studies in a reliable way and are often used to inform policy and practice, such as guideline development [1, 2]. Whilst they are often associated with medical research and randomised controlled trials, they can be used to address any research question using any relevant type of research [3]. A critical feature of a systematic review is the application of scientific methods to uncover and minimise bias and error in the selection and treatment of studies [4, 5]. However, the large and growing number of published studies, and their increasing rate of publication, makes the task of identifying relevant studies in an unbiased way both complex and time consuming [6].

In order to minimise the impact of publication bias [7], reviewers make efforts to identify *all* relevant research for inclusion in systematic reviews. This has always been a challenging and time-consuming aspect of reviewing, but the challenge is growing due to the increase in the number of databases to search and the number of papers and journals being published; moreover, as recent work has suggested that there is an inbuilt North American bias in many major bibliographic databases (e.g. PubMed), a wide range of smaller databases needs to be searched in order to identify research for reviews that aim to maximise external validity [8]. In practice, this means adopting a multi-layered approach to searching which combines: extensive Boolean searches of electronic bibliographic databases, specialised registers and websites; with individual approaches to authors and key informants; and the following of ‘citation trails’ (identifying which papers are cited by a relevant study and which papers in turn cite the paper that it is reported in) [9]. Of these three approaches, searching databases yields around three quarters of the studies finally included [10].

Unfortunately, the specificity of sensitive electronic searches of bibliographic databases is low (for definitions of specificity, recall and other key metrics, see Table 1). Reviewers often need to look manually through many thousands of irrelevant titles and abstracts in order to identify the much smaller number of relevant ones [7]; a process known as *screening*. Reviews that address complex health issues or that deal with a range of interventions (e.g. a typical public health review might be concerned with ‘interventions to promote physical activity’) are often those that have the most challenging numbers of items to screen. Given that an experienced reviewer can take between 30 seconds and several minutes to evaluate a citation [11], the work involved in screening 10,000 citations is considerable (and the screening burden in some reviews is considerably higher than this) (see also [12]).

**Table 1:** **Definitions of performance measures reported in the studies**

Measure	#	Definition	Formula
*Recall (sensitivity)*	22	Proportion of correctly identified positives amongst all *real* positives	
*Precision*	18	Proportion of correctly identified positives amongst all positives.	
*F measure*	10	Combines precision and recall. Values of *β* < 1.0 indicate precision is more important than recall, whilst values of *β* > 1.0 indicate recall is more important than precision	Where *β* is a value that specifies the relative importance of recall and precision.
*ROC (AUC)*	10	Area under the curve traced out by graphing the true positive rate against the false positive rate. 1.0 is a perfect score and 0.50 is equivalent to a random ordering	
*Accuracy*	8	Proportion of agreements to total number of documents.	
*Work saved over sampling*	8	The percentage of papers that the reviewers do not have to read because they have been screened out by the classifier	
*Time*	7	Time taken to screen (usually in minutes)	
*Burden*	4	The fraction of the total number of items that a human must screen (active learning)	
*Yield*	3	The fraction of items that are identified by a given screening approach (active learning)	
*Utility*	5	Relative measure of burden and yield that takes into account reviewer preferences for weighting these two concepts (active learning)	Where β is the user-defined weight
*Baseline inclusion rate*	2	The proportion of includes in a random sample of items before prioritisation or classification takes place. The number to be screened is determined using a power calculation	Where *n*_*i*_ = number of items included in the random sample; *n*_*t*_ = total number of items in the random sample
*Performance (efficiency)* ^*a*^	2	Number of relevant items selected divided by the time spent screening, where relevant items were those marked as included by two or more people	
*Specificity*	2	The proportion of correctly identified negatives (excludes) out of the total number of negatives	
*True positives*	2	The number of correctly identified positives (includes)	TP
*False negatives*	1	The number of incorrectly identified negatives (excludes)	FN
*Coverage*	1	The ratio of positives in the data pool that are annotated during active learning	Where *L* refers to labelled items and *U* refers to unlabelled items
*Unit cost*	1	Expected time to label an item multiplied by the unit cost of the labeler (salary per unit of time), as calculated from their (known or estimated) salary	time_expected_ × cost_unit_
*Classification error*	1	Proportion of disagreements to total number of documents	100 % − accuracy %
*Error*	1	Total number of falsely classified items divided by the total number of items	
*Absolute screening reduction*	1	Number of items excluded by the classifier that do not need to be manually screened	TN + FN
*Prioritised inclusion rate*	1	The proportion of includes out of the total number screened, after prioritisation or classification takes place	Where n_ip_ = number of items included in prioritised sample; n_tp_ = total number of items in the prioritised sample

TP = true positives, TN = true negatives, FP = false positives, FN = false negatives.

^a^Performance is the term used by Felizardo [13], whilst efficiency was used by Malheiros [14].

[Not used in the included studies, though worthy of note is the ‘G-mean’. This is the geometric mean of sensitivity and specificity, and it is often used for a metric alternative to F score in evaluating classification on imbalanced datasets. G-mean evaluates the classification performance for classification labels, whilst AUC evaluates the classification performance for classification scores. Note that these metrics alone do not always reflect the goal in systematic reviews [15].

Reviewers are thus faced with two competing demands. Reviews that are to be used to inform policy and practice often need to be completed to externally defined (often short) timetables within limited budgets; but in order for a review to be an accurate reflection of the state of knowledge in a given area, it needs to be comprehensive.

The need to complete reviews to tight timescales has led (particularly in health technology assessments and other rapid reviews) to the adoption of highly pragmatic (and relatively *specific*) strategies to searching in order to limit the number of studies to screen—even though relevant research is probably missed because of this [16]. Limiting the recall of a search may undermine one of the most important principles of a systematic review: that its results are based on an unbiased set of studies. The key problem—which this paper aims to begin to address—is that there are currently no widely accepted alternative ways of dealing with this issue. Reviews are at risk of either limiting their searches to such a degree that the validity of their findings is questionable or of increasing the time and resources they require and thus risk being unable to inform policy and practice.

### Proposed ‘solution’: the (semi)-automation of screening

Broadly speaking, text mining is defined as the process of discovering knowledge and structure from unstructured data (i.e., text) [17, 18]. In the context of finding research for inclusion in a review, we are interested in automated techniques of discovering whether a given study (described by a title and abstract) is relevant to our review [19, 20]. There are two ways of using text mining that are particularly promising for assisting with screening in systematic reviews: one aims to prioritise the list of items for manual screening so that the studies at the top of the list are those that are most likely to be relevant; the second method uses the manually assigned include/exclude categories of studies in order to ‘learn’ to apply such categorisations automatically [19]; whilst the technologies to perform each may be similar, we separate them here as they are conceptually distinct. The prioritisation of relevant items may not appear to reduce workload (if all citations are to be screened manually anyway), but when there are large numbers of studies to screen manually, identifying most of the relevant ones quickly enables some members of a reviewing team to begin the next stages of the review, whilst the remainder of mostly irrelevant citations are screened by other team members. This reduces the time from review commencement to completion, even if the total workload remains the same.

By reducing the burden of screening in reviews, new methodologies using text mining may enable systematic reviews to both: be completed more quickly (thus meeting exacting policy and practice timescales and increasing their cost efficiency); AND minimise the impact of publication bias and reduce the chances that relevant research will be missed (by enabling them to increase the recall of their searches). In turn, by facilitating more timely and reliable reviews, this methodology has the potential to improve decision-making across the health sector and beyond.

### The research problem

Whilst the logic behind applying text mining to the screening stage of systematic reviews has intuitive appeal, there are obvious concerns that might be raised by the systematic review community [21]. Firstly, there is not a lot of information about text mining written for systematic review audiences. The vast majority of papers on this topic are produced by computer scientists in journals and conference proceedings in the field of medical informatics or artificial intelligence. This means that they are not particularly accessible to systematic reviewers who need to make decisions about their review processes, both in terms of the level of technical detail presented in the reports and in the exposure such papers would have in systematic review communities.

Secondly, for these technologies to achieve broad uptake, they should be accessible to systematic reviewers without the need for a computer scientist to write bespoke code or undertake custom processing of text for individual reviews. Specialist advice may be required, but it should be akin to the need for occasional specialist statistical advice, rather than being at the level of operating the text mining tools. Any implementation issues need to be identified and resolved before rolling such technologies out to the intended users.

Thirdly, there are various ways in which workload could be reduced through these technologies (reducing number needed to screen; text mining as a second screener; increasing the rate (speed) of screening and improving workflow through screening prioritisation). However, not all technologies allow all types of workload reduction to be achieved. In order to make informed decisions about using such technologies, systematic reviewers need to know which technologies can be used for which workload reduction goal.

Fourthly, systematic reviews are a relatively new area in which text mining technologies have been applied. Some of the assumptions of text mining technologies in other applications do not hold when transferred to the review context. For instance, systematic reviewers generally place strong emphasis on high recall—that is, a desire to identify all the relevant includable studies—even if that means a vast number of irrelevant studies need to be considered to find them. When applied in other areas, precision (reducing the number of irrelevant items) and accuracy (correctly classifying items as relevant or irrelevant) are typically more valued. To be acceptable to the systematic review community, new technologies must address the particular challenges and demands of this context (We should also note at this point that we have no guarantee of perfect recall even with current methods, as search strategies are tailored to the resource available to screen results, and humans are likely to make mistakes during their manual sifting through records.).

Finally, the methods, their relative success and the metrics used to evaluate them have not yet been pulled together in a systematic way; this current study aims to fill that research gap.

### Aims and research questions of the review

The primary aim of this review is to gather and present the available research evidence on existing methods for text mining related to the title and abstract screening stage in a systematic review, including the performance metrics used to evaluate these technologies^a^. The purpose of this is to inform systematic reviewers of the current state of text mining methods for use in reducing workload at the screening stage, with a consideration of the potential benefits and challenges when implementing such technologies. Whilst we have explored the more technical aspects of text mining technologies in our data extraction, the intended audience of this paper are users of the technologies rather than computer scientists, and so technical issues are largely dealt with at a conceptual level.

Following directly from the research problem as delineated above, we looked to answer the following questions:

- What is the state of the evidence base related to automating (or semi-automating) the screening stage (based on titles and abstracts) of a systematic review? Specifically,
- What methods are available; and
- How has the field developed over time?
- How has the workload reduction issue been evaluated? Specifically,
- What has been compared, using what research study designs?
- What metrics are available for evaluating the performance of the approaches?
- What are the stated purposes of (semi-)automating the screening stage through text mining in terms of workload reduction, what types of methods have been used to address each purpose, and how effective were they?
- How, and with what effect, have key contextual problems of applying text mining to systematic review screening been addressed, specifically as relates to the following challenges:
- The importance of high recall for systematic reviews?
- The risk of hasty generalisation when training from a certain pool of known includes and excludes?
- The problem of imbalanced datasets, in which there are typically many more excludes than includes?
- Applying the technologies to review updates?
- What challenges to implementation emerge from reviewing the evidence base?

## Methods

We conducted a systematic review of research papers on applications of text mining to assist in identifying relevant studies for inclusion in a systematic review. The protocol can be sent on request by the authors.

### Information management

All records of research identified by searches were uploaded to the specialist systematic review software, EPPI-Reviewer 4, for duplicate stripping and screening [22]. This software recorded the bibliographic details of each study considered by the review, where studies were found and how, reasons for their inclusion or exclusion, descriptive and evaluative codes and text about each included study, and the data used and produced during synthesis.

### Search methods

Database and website searches were conducted in December 2013. Sources were searched from 2005 onwards. This date was chosen because, according to Jonnalagadda and Petitti [23], the first proposed application of text mining to screening in systematic reviews was in 2005 (though this was not an evaluation of a method and so was not included in our review).

Details of the electronic search strategy, including databases searched and terms used, can be found in Additional file 1: Appendix A; the PRISMA flow diagram can be viewed in Additional file 2: Flow diagram.

We also included papers known to the team and as recommended by colleagues. We checked the reference lists of all included studies for additional relevant studies. We also followed forward citation recommendations in Science Direct. A cut-off for identifying studies for inclusion in the review was set at 28 February 2014.

After all searches were completed, 1,253 records were identified. These were screened for relevance to our review using the inclusion criteria outlined below.

### Inclusion criteria

Studies were screened in a two-stage screening process. First, records were assessed against the following criteria based on their titles and abstracts:

1. Must be published after 2004
2. Must be relevant to text mining
3. Must be relevant to the screening (document selection) stage of a systematic review (or a review of the evidence that follows systematic principles, such as health technology assessment (HTA) or guidelines development)

After an initial piloting of the first stage criteria to establish common understanding of the criteria, records were screened once by two researchers (AOM and JT) who are familiar with systematic reviewing and text mining methods. Any records of doubtful relevance were marked with a ‘query’ tag and discussed by the two researchers until agreement was met (Agreement was always reached, and so recourse to a third reviewer was not required.).

The full-text documents of records that met these criteria (*n* = 69) were retrieved and proceeded to the second stage of screening. The criteria for assessing the full-text documents were:

1. Must be relevant to text mining methods or metrics
2. Must be relevant to the screening stage of a systematic review (or similar evidence review)
3. Must not be a general discussion of the use of text mining in systematic reviewing screening. That is, the record must present a detailed method or evaluation of a method.

The second stage of screening was conducted by one researcher (AOM), with queried records checked by the second researcher (JT) (reviewer agreement was 100% at this stage). After full-text screening, a total of 44 records were identified as relevant to the review questions.

### Data extraction

Data extraction was conducted by one researcher (AOM) and checked for accuracy and completeness by a second researcher (JT) and discrepancies resolved by a second check and/or discussion. We extracted and recorded information on the following broad issues (see Additional file 1: Appendix B for the full data extraction tool, Appendix C for the list of studies included in the review and Appendix D for the characteristics of included studies):

- ● Bibliographic details
- ● Evaluation context (details of review datasets tested)
- ● Evaluation of active learning (if applicable) (see below for definition)
- ● Evaluation of classifier
- ● Evaluation of feature selection
- ● Implementation issues
- ● About the evaluation (the methodology and metrics used)
- ● Study type descriptors
- ● Critical appraisal
- ● Comments and conclusions

Extraction consisted of two types of data: direct quotations from the papers, which were gathered through line-by-line coding of the papers; and categorical data, which were gathered by noting the presence or absence of certain characteristics. These two types of data were collected simultaneously. For example, a tick box was checked if a study reported using a support vector machine (SVM) classifier, and line-by-line coding of text that described the SVM was associated with that tick box in the EPPI-Reviewer 4 software [22].

### Synthesis methods

The reviewers discussed the key issues that needed to be covered in the review, as well as themes that had emerged through extracting data from the studies. On that basis, an outline structure for the synthesis was developed. Under the outline subheadings, a narrative was developed that drew on both the line-by-line coded text and the categorical data. The categorical data allowed for the generation of frequency tables and cross tabulations that described the state of the evidence base; whilst the coded text allowed for a richer interrogation of the emerging themes.

## Results

The results are presented in order of the research questions posed. Since some issues raised apply beyond the systematic review context, which limited the range of papers about text mining that we formally included, we have inserted some commentary (entitled ‘further information on this topic’) where information from other domains may illuminate a specific issue.

### Development of the evidence base

In this section, we address research question 1: *What is the state of the evidence base related to automating (or semi-automating) the screening stage (based on titles and abstracts) of a systematic review?*

### Chronological developments

**Figure 1:**
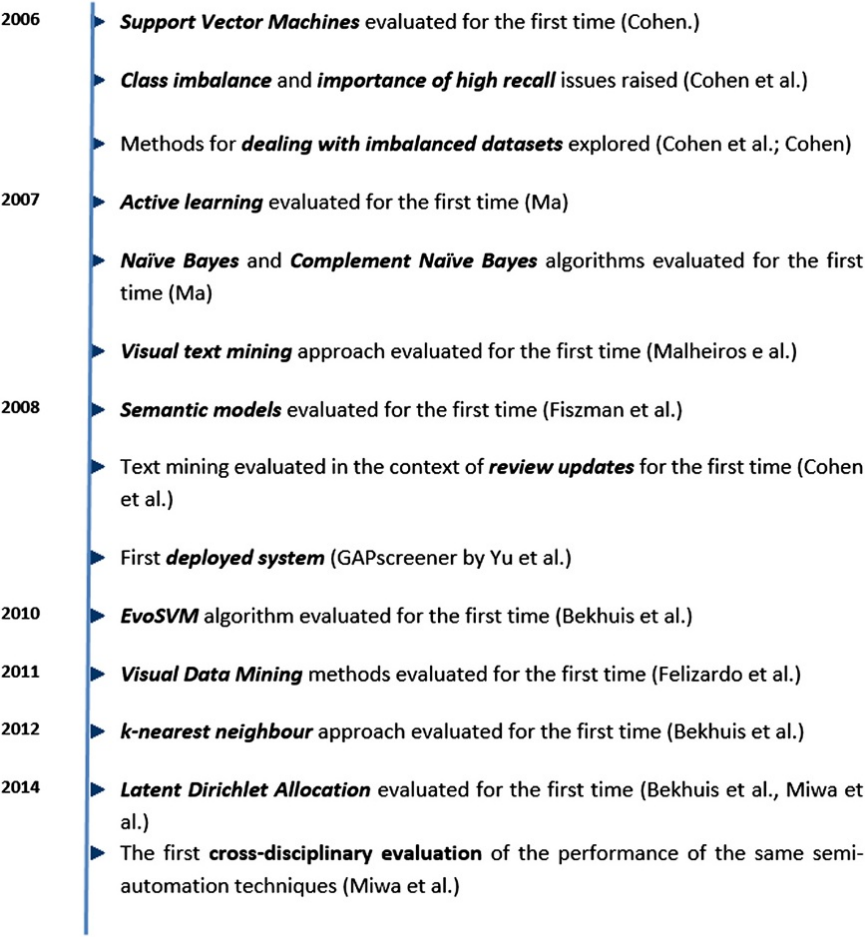
**Brief timeline of developments in the use of text mining technologies for reducing screening burden in systematic reviews.**

Our 44 included studies fall within the 8 years between January 2006 and January 2014—an average of 5.6 evaluations a year. As can be seen in the timeline presented in Figure 1, almost every year saw the evaluation of a newly applied type of classifier or some new consideration of the application of text mining to screening. Indeed, most papers present a new ‘twist’ that distinguishes it from those before, with very few replications or comparisons between papers. The developments highlighted in the timeline are those which we had defined *a priori* in our data extraction tool and therefore also how the synthesis below is structured; they should therefore be considered to be indicative of interesting developments, rather than being a comprehensive list of every innovation (For example, also worthy of note are the decision trees by Frunza and colleagues in 2010 [24]; and dual supervision and elicited utility by Wallace et al. (also in 2010 [25])).

This suggests a rapidly evolving evidence base (It also has implications for the later parts of this synthesis, as it is difficult to come to any overarching conclusions about which approach works best.).

### Workload reduction approaches

In this section, we address research question 2: *What are the stated purposes of (semi-)automating the screening stage through text mining in terms of workload reduction, and what types of methods have been used to address each purpose?*

It is evident from the literature that there are several possible ways to reduce screening workload. The approaches that have received attention in terms of text mining are: reducing the number of items that need to be screened manually; reducing the number of people needed to screen the items; increasing the rate (or speed) of screening; and improving workflow. Table 2 shows the number of studies that implicitly or explicitly addressed each of these approaches. Each of these will be discussed in turn.

**Table 2:** **The number of studies implicitly or explicitly addressing screening workload problems (** ***n*** **= 44)**

Workload reduction approach	Number of studies
Reducing number needed to screen	30
Text mining as a second screener	6
Increasing the rate (speed) of screening	7
Improving workflow through screening prioritisation	12

*Note.* Some studies adopted more than one approach to workload reduction, so column total is greater than 44 studies.

### Reducing the number of items that need to be screened

In many reviews, the number of items to be screened is very large. For example, 4 out of the 31 Cochrane Collaboration systematic reviews published in March 2014 had over 10,000 items to screen [26–29]. This can be a particular problem for searches for certain types of study designs, such as is the case with searches for non-randomised controlled trials, for which database filters are not available or consistently used [30]. Large numbers of items to screen is even more evident in non-clinical disciplines, in which search strategies tend to be broader in response to broader research questions, less precise or consistent terminology and the lack of controlled vocabularies; for example, EPPI-Centre reviews on topics in public health, education and social care regularly exceed 20,000 items to be screened. At its most extreme, one review identified upward of 800,000 items and another over 1 million items to be screened (see [31] for a description of such ‘extreme reviewing’). Given that an experienced reviewer can take between 30 seconds and several minutes to evaluate a citation [11], the work involved in screening even as ‘few’ as several thousand citations is considerable.

An obvious solution to reducing workload is therefore to reduce the number of items that need to be screened manually. Historically, the volume of records returned from a search was determined in part through the search strategy: the number of records identified could be reduced either through searching fewer sources or through carefully constructed database queries. The latter approach usually adopted an emphasis on the precision of the search over its recall. However, some method guidelines specifically recommend favouring recall over precision in order to avoid missing relevant studies (e.g., the Campbell Collaboration’s guide to information retrieval and the US Institute of Medicine of the National Academies [32, 33]).

Therefore, resource-efficient approaches that maximise recall are needed, and a number of different models have been identified here. The vast majority of studies included in the review (*n* = 30) implicitly or explicitly propose using text mining for the purpose of reducing the number of studies that need to be screened manually. Within this set of studies, there are two main approaches to excluding items from a review. The first approach is to use a *classifier* that makes explicit in/out decisions; 23 studies evaluated this approach [11, 14, 23, 25, 34–51]. The second approach is to use a *ranking or prioritisation system* and then exclude items that fall below some threshold or criterion, or that lie within a ‘negative prediction zone’ [31, 52–57]; seven studies used this approach. Whilst many classifiers employing the first approach inherently assign some kind of score that indicates confidence in how likely an item is to be an include or exclude (akin to the ranking in the second approach), this is usually ‘hidden’ from the reviewer such that the decisions are presented as complete. In contrast, the second approach may require a reviewer to continue manual screening until the (reviewer-specified) criterion is met.

It is important to note that the final approach, *active learning*, can fit loosely into both of the abovementioned camps. Active learning (evaluated in nine studies [11, 23, 25, 31, 40, 45, 48, 49, 58]) is an iterative process whereby the accuracy of the predictions made by the machine is improved through interaction with reviewers. The reviewer—or review team—provides an initial sample of include/exclude decisions that the machine ‘learns’ from; the machine subsequently generates a ranked list and requests the reviewer to provide decisions on items high in the list that it will learn the most from. The machine adapts its decision rule including the information from the additional items and generates a new list of items for the reviewer to screen. This process continues, with the number of reviewer decisions growing and a greater number of relevant items found than would otherwise be the case, until a given stopping criterion is reached and the process ends. Although the final include/exclude decisions for any items not screened manually come from the classifier, the human screener still has some control over the training process and the point at which manual screening ceases.

In all cases, authors reported that the systems tested led to a reduction in workload; however, given the diversity of approaches and the lack of overlap (replication) between evaluations, it is impossible to conclude whether one approach is better than the other in terms of performance. Typical performance reported a reduction in manual screening workload from less than 10% (e.g. [41]) up to more than 90% (e.g. [48]). Where expressed as a workload reduction, studies tended to report reductions of between approximately 40% and 50% of work saved (e.g. [25, 40, 41, 55]). Studies differed from one another in terms of the recall that they aimed for. Some expressed results in terms of 95% recall (e.g. [23]), whereas others expressed their results in terms of retrieving all relevant studies (e.g. [48]). Razavi and colleagues took a critical perspective with regard to manual decisions too, concluding that ‘Since the machine learning prediction performance is generally on the same level as the human prediction performance, using the described system will lead to significant workload reduction for the human experts involved in the systematic review process’ [44].

### Text mining as a second screener

Methods guidance for conducting systematic reviews often suggests that more than one person should screen all (or some proportion) of the records returned by the searches (e.g., the Institute of Medicine (Washington, DC) states in Standard 3.3.3. ‘Use two or more members of the review team, working independently, to screen and select studies’ [33]). The rationale behind this approach is that a single screener can inadvertently introduce bias into the study selection process either because of their interpretation of the inclusion criteria or through their understanding of the content of titles and abstracts. Moreover, given the volume of records to be reviewed, it is conceivable that some relevant records might ‘slip through the net’. It is believed that if there is consistency in the inclusion decisions amongst two or more independent screeners, then the screening process is not likely to be biased. This, however, becomes a very labour-intensive process—particularly when the number of records to screen is high. Although some guidance suggests that if sufficient inter-reviewer reliability is achieved that it is acceptable to ‘double screen’ only a proportion of the records when there is a large number to screen, this still can add a substantial amount of resource to an already time-consuming procedure.

To combat this workload issue, six papers have advocated the use of text mining as a second screener: replacing or supplementing the additional human reviewer that would be required at this stage [24, 30, 59–62]. In this model, one human reviewer screens all of the records and the machine acts as the independent check (or presents a vastly reduced list of items to be screened to an additional human reviewer). The evaluations of workload reduction in this area have all been on a classifier model, in which explicit in/out decisions are made by the machine. Results from the evaluations are positive—the classifiers had good agreement with the human reviewer/s. Three of these papers were authored by Bekhuis and colleagues [30, 59, 60], who report that their approach could reduce manual workload by between 88% and 98% [60]. Frunza and colleagues report two studies in this area [24, 61] and Garcia one study [62]. Like Bekhuis, they report positive results from their evaluations, though they present their findings in terms of high recall rather than workload reduction, and so a direct comparison cannot be made.

### Increasing the rate of screening

An alternative approach to those above, which emphasises reducing the number of items that need to be screened manually, is to aid researchers in coming to a decision about each item more quickly; that is, to increase the rate of screening. To achieve this, *visual data mining* (VDM) approaches attempt to create a visual representation of the connections between documents (using term similarity and/or author connections) to assist the screener in identifying studies easily that are more likely to be similar to each other. Thus, once a relevant document is identified, they can quickly scan other documents that appear to be similar to the relevant document (and similarly, identify documents that are likely to be excluded quickly). The approach assumes that humans can make a decision about a study’s relevance faster using this additional visual information than relying on the textual information in the titles and abstracts alone [13].

Five evaluations of visual data mining were identified [13, 14, 63–65], all in the field of software engineering. The evaluations of visual data mining differ from evaluations of other text mining approaches in that they employ a controlled trial evaluation design to compare the speed and accuracy with which a human can screen items using VDM or without using VDM. The results suggest that humans can screen faster with VDM aids than without, although the accuracy of the human screeners does not appear to change substantially [13, 14, 63–65].

A second approach to speeding up the rate of screening that is embedded within approaches to reducing the number needed to screen is through *efficient citation assignment*. The only example that was identified of this type was by Wallace and colleagues [49]. In that paper, the authors emphasise that most review teams have a combination of expert and novice screeners. Within the context of an active learning approach, they developed an algorithm that incorporates both information about the relevance of each item and the expected time that it will take to annotate that item; on that basis, the algorithm selects citations specifically for expert and novice reviewers to label. The authors reported that this approach enabled more items to be screened in the same amount of time compared with typical active learning approaches.

### Improving workflow efficiency through screening prioritisation

Screening prioritisation is ultimately a form of efficient citation assignment, in that it aims to present reviewers with an ordered list of the items, with the items that are most likely to be relevant to their review at the top of the list. However, it differs from the model described by Wallace et al. [49] in that it is not necessarily embedded within an approach that is attempting to reduce the number needed to screen and it does not differentially assign items to different types of reviewers (i.e., experts versus novices).

There are various proposed benefits of this approach to workflow efficiency. One is that reviewers gain a better understanding of the inclusion criteria earlier in the process, as they encounter more examples of relevant studies sooner than would otherwise be the case. It also enables the retrieval of the full text of documents to start sooner than can occur when citations are screened essentially at random. This can be important, as obtaining the full-text reports brings forward their full-text screening, the checking of their bibliographies and, critically, enables contact to be made with study authors much earlier in the review. It is also possible that this will make the screening process faster, once the vast majority of relevant studies are identified, as the screeners become more confident that items later in the list are less likely to be relevant. This could also help with the problem of over-inclusiveness that is often experienced in reviews, in which reviewers tend to be cautious and include many more items at this early stage than ultimately make it into the review.

Cohen highlighted another potential benefit: ‘In reviews with searches that result in a large number of citations to be screened for retrieval, reviewing the documents in order of their likely importance would be particularly useful. The remainder of the citations could be screened over the following months, perhaps by the members of the team with less experience, whilst the work of reviewing the includable studies is ongoing’ ([66] p. 692) (An ongoing project at the EPPI-Centre, which had a large volume of items to be screened (>38,000) but with a very tight timeframe, has taken advantage of this benefit [67].).

There are also potential benefits for review updates. Cohen stated that ‘by reviewing the most likely important documents before other documents, the human reviewers or curators are more likely to be able to “get up to speed” on the current developments within a domain more quickly’ ([68] p. 121). In quite a different application of text mining to the screening process, Cohen later explored the use of prioritisation for identifying when a review update was required, which would involve sending alerts to the review team when likely relevant new studies are published [69].

In other words, this approach emphasises improving workflow in a review and has proposed benefits for efficiency beyond reducing workload in the title and abstract screening phase. Four studies adopted a prioritisation approach to improve workflow [58, 66, 68, 69]. All four evaluations reported benefits of this approach.

Note that screening prioritisation can also be used to reduce the number of items needed to be screened if a screening cut-off criterion is established (see section on this workload reduction approach, above). Seven studies that have used screening prioritisation did so to reduce the number needed to screen and reported benefits in terms of the amount of work saved [31, 52–57]. (Again, the metrics and processes varied, so it is not possible to estimate overall or mean statistics across these studies).

### Specific issues relating to the use of text mining in systematic reviews

In this section, we address research question 3: *How have key contextual problems of applying text mining to systematic review screening been addressed?* These reflect the challenges that need to be addressed when applying methods developed for other applications to the case of systematic review screening.

### The importance of high recall for systematic reviews

As mentioned in the ‘Background’ section, recall is often prioritised over precision in systematic reviews. This is because it is generally considered to be critical to retrieve all relevant items to avoid biasing the review findings. The importance of high recall of relevant studies is likely to be critical in the acceptability and uptake of text mining techniques by the systematic review community. Indeed, the authors of one paper reflected that ‘If those who rely on systematic review to develop guidelines and policy demand 100% recall and informatics approaches such as ours are not able to guarantee 100% recall, the approaches may be doomed’ ([23] p. 15).

Many of the studies in this review explicitly refer to the importance of high recall and the implications it might have for text mining applications in this area (studies which discuss the importance of high recall include [11, 23, 24, 30, 38, 40, 41, 44, 48, 49, 53, 54, 58, 60, 61, 70]). However, few of the studies directly built into the technology an approach to maximising recall. Those that did directly attempt to maximise recall are discussed below.

#### Voting or committee approaches for ensuring high recall

One approach to ensuring that studies are not missed is to use a voting or committee approach. Essentially, multiple classifiers are run simultaneously, and then a ‘vote’ is taken on each item to determine whether it is likely to be relevant or not. A conservative approach would be to put forward for human screening any item that receives at least one ‘include vote’ (e.g., Wallace et al. [11]); an approach that places additional emphasis on precision might set a minimum number of agreeing votes (e.g., >50% of the classifiers must agree that an item is an include [44]).

The appeal of such approaches is that the classification decision is less susceptible to missing studies that do not resemble the training set of includes, because each classifier can start with a different training set. Several studies have used this approach, with different numbers of classifiers used in the committee. Razavi used a committee of five classifiers [44]; Wallace and Frunza used (up to) eleven classifiers [11, 24, 61]; Ma used two classifiers [40]. Only Frunza has considered whether the number of votes makes a difference, as discussed below [24, 61].

In Frunza (2010), if at least one decision for an abstract was to include it in the systematic review, then the final label was ‘Included’ [24]. They then tested whether the number of votes (i.e., number of classifiers) made a difference to recall and precision. They concluded that the 2-vote technique is superior to the other voting techniques (1-vote, 3-vote, 4-vote) in terms of the F measure and work saved over sampling (WSS). The highest level of recall was achieved through the 4-vote technique. The success of combined human-machine screening was similar in their later study [61], with the conclusion that the 2-vote technique was the best performer. Importantly, Frunza noted that precision decreased slightly when the human decisions were added to the machine decisions (i.e., the human incorrectly included some items). This might be relevant to the observation that human screeners tend to be over-inclusive (discussed in a later section).

(We will return to the issue of ‘voting’ approaches below, in the section on ‘Hasty generalisation’).

#### Specialist algorithms

At least three types of classifiers have been modified to include a specialist algorithm that adjusts the learning rate of the classifier to penalise false negatives. Cohen et al. applied a ‘false negative learning rate’ to their voting perceptron classifier expressing this as a ‘cost-proportionate rejection sampling’ strategy [36]. Matwin et al. added a heuristic weight factorization technique to their complement naïve Bayes (CNB) algorithm to maximise recall when their original algorithm had unacceptably low recall (<95%) [41]. Bekhuis also modified a complement naïve Bayes classifier by optimising the decision parameters using F3: a summary measure of performance that overweights recall relative to precision [60]. Wallace and colleagues modified their support vector machine approach to penalise more severely for false negatives compared with false positives [48].

All of these studies were retrospective evaluations in which the performance of a classifier was compared against completed include decisions and all reported good results in terms of recall and workload reduction. Future evaluations of this approach should consider whether the amount and/or quality of the training data make a difference to the ability of these modifications to adequately penalise false negatives. The reason for this is that, if used in a ‘live’ review, there might be only a small number of human-labelled items in the training set to be able to determine whether the classifier has incorrectly rejected a relevant study. If there are only a small number of includable studies in the entire dataset, then such penalties might not be implementable.

#### Human input

Ma proposed using active learning as a method for assuring high recall [40]. The logic behind this is that the algorithm continues to ‘learn’ as more items are manually screened and so the decision rule is adaptable and less reliant on the initial training set. However, Ma’s [40] results suggest that recall actually declined when active learning was added to a support vector machine or decision tree classifier and made no difference to the recall of a naïve Bayes classifier. Further research on this is needed to determine why this might be the case.

### Hasty generalisation

The term ‘hasty generalisation’ refers to a bias which can occur because the features in the training set are not representative of the population; as opposed to other forms of ‘biased training sets’ (e.g. where bias occurs from non-randomised sampling). If the initial training set of documents in a systematic review is not fully representative of the range of documents which are of interest, it is possible that these documents will be missing from the set of studies identified as relevant through automation (see [25]). To exclude relevant studies due to their use of different terminology from those that are included would be to inject a systematic bias which would be unacceptable in the vast majority of reviews.

Several methods for dealing with this have been evaluated or discussed: drawing on reviewer domain knowledge, using patient active learning methods and employing an ensemble of classifiers that vote on whether an item should be included or not. These are elaborated on in the following sections.

#### Reviewer domain knowledge

Some studies evaluated or discussed drawing on the knowledge of the human reviewers to play a part in the text mining process. This is particularly suited to active learning approaches. Jonnalagadda and colleagues suggested that, in active learning, ‘the dynamically changing query set, which decides which document will be presented next, could be easily modified at any stage by removing or adding terms to the query set. In this way, the possibility of not finding documents that use different words could be further minimised by allowing active participation of the users in defining the terms in the query set’ ([23] p. 15). They did not, however, test this approach empirically.

In addition to other text mining methods, Shemilt et al. employed an approach that used ‘reviewer terms’ (terms specified by the review team as being indicative of an includable or excludable study) [31]. The text contained in each title-abstract record that was yet to be screened was analysed and the number of relevant and irrelevant terms they contained was calculated. A simple ratio of these values was then generated, and items were ranked according to this ratio. The authors argue that ‘The purpose of this method is to act as a counterpoint to the automated technologies; whereas in ATR [automatic term recognition] and AC [automatic classification], the results are heavily determined by those studies already identified as being relevant; RT [reviewer terms] offers another perspective on potential relevance, offering some protection against the problem of hasty generalization’ ([31] p. 45). This might offer reassurance to review teams that no relevant items are being erroneously discarded and is an easy approach to implement if the reviewers are familiar with the key terminology.

A more holistic approach was evaluated by Wallace et al. [25]. As in Shemilt et al. (above), reviewers provided terms that were indicative of includes and excludes (although the terms were ranked in order of ‘indicativeness’ in the Wallace paper). Wallace et al. suggested that combining prior reviewer knowledge with the machine model could be more effective at avoiding hasty generalisation and tested a variety of combinations in terms of the timing at which the reviewer knowledge rankings were emphasised relative to the machine labelling. They concluded that beginning with a bias towards the reviewer rankings and subsequently decreasing its importance as labelling proceeds would be the most effective way of combining reviewer domain knowledge in the process; however, they also noted ‘How this should be done precisely remains a problem for future work’ ([25] p. 8).

In addition, in a study which came to light after our formal searches were complete, Small et al. utilised reviewer ‘labelled features’ within what they called a ‘constrained weight space SVM’ [71]. They found that, by allowing reviewers to influence the decisions made by the classifier, it is possible to obtain better results with smaller samples of training records.

#### Patient active learning

‘Patient active learning’ was first proposed by Wallace et al. as a means of overcoming hasty generalisation using an active learning approach [11]. The distinguishing feature of ‘patient’ active learning is that training is based on different ‘views’ of the records (e.g. classifiers based on titles or abstract or MeSH terms) which are selected at random at each iteration of the active learning process. The additional variability that this approach injects into the process above the use of a single ‘view’ aims to ensure that the system as a whole is exposed to as wide a variety of relevant studies as possible and thus does not overly narrow the range of items it considers to be relevant.

Wallace and colleagues evaluated four different active learning strategies and found that patient active learning outperformed the others [11]. In a study which replicated some of Wallace’s work on the same data, Miwa and colleagues evaluated a range of active learning enhancements and found that patient active learning is certainly better than some strategies, though not as good as others [45].

#### Voting or committee approaches for dealing with hasty generalisation

The concept of a committee of classifiers was earlier introduced for helping to ensure high recall. Given that hasty generalisation would logically lead to lower recall, it is unsurprising that this approach has also been suggested as a solution to hasty generalisation.

Two studies explicitly refer to this approach. Miwa et al. reported that voting showed some improvement over non-voting approaches, especially for one particularly ‘messy’ dataset with respect to the terminology used in that review topic [45]. Shemilt et al. did not compare voting with non-voting approaches but ran the classifier multiple times and then manually screened only those items that were consistently classified as being relevant [31]. This approach seems likely to have increased precision at the expense of sensitivity.

### Dealing with imbalanced datasets

At the title and abstract screening stage of a typical systematic review, the dataset is imbalanced in that there are usually far more excluded studies than included studies. One paper reported a median search precision (number of included studies divided by total number of items located through searching) of 2.9% across 94 health-related systematic reviews [72]. This translates to an imbalance in which there are approximately 33.5 times as many excludes as includes. Search precision can be much less than this, resulting in even greater imbalances.

In text mining evaluations, this is referred to as the ‘class imbalance’ problem (where ‘class’ refers to the designation as an include or an exclude). It is a problem for text mining as there are far fewer relevant items compared to non-relevant items on which to train the classifier or text mining technology. Also, Wallace et al. state that ‘class imbalance presents a problem for classification algorithms, because they have typically been optimised for accuracy, rather than the recall of a particular class’ ([11] p. 5). Since it is possible to have high accuracy even if a system produces many false negatives [73], this could be a problem for systematic reviews where missing relevant studies is highly undesirable.

To counter the class imbalance, various methods have been proposed. They generally rely on up-weighting the number of includes or down-weighting the number of excludes; or undersampling the number of excludes used in the training set. The various approaches are described in the following sections.

#### Weighting

Weighting approaches assign greater weights to positive instances (includes) than to negative instances (excludes). Generally, the weight is set to the ratio of the number of positive instances to the number of negative instances.

Compared to an un-weighted method or an aggressive undersampling method (described below), Miwa et al. reported better performance of active learning models on a variety of imbalanced datasets [45]. This was particularly the case when weighting was used in conjunction with a ‘certainty’ approach, in which the next items to be annotated in the active learning process were selected because they had the highest probability of being relevant to the review, based on the output of classifiers trained on previously annotated items.

Cohen et al. also reported good results for a weighted model, in which they modified their voting perceptron classifier to incorporate a false negative learning rate (FNLR) [36]. Across 15 reviews, they found that the FNLR should be proportional to the ratio of negative to positive samples in the dataset in order to maximise performance.

#### Undersampling

Undersampling involves using fewer non-relevant studies in the training set than might be expected given their prevalence in the entire dataset. Two different types of undersampling have been tested in this context: random and aggressive.

*Random undersampling* involves randomly selecting a training set with the same number of relevant and non-relevant studies. This approach was adopted in four studies that did not compare random undersampling to other methods for dealing with class imbalance [11, 31, 39, 48].

Ma compared five undersampling methods with their active learning naïve Bayes classifier—one of which was random undersampling [40]. Method 1 involved selecting the negative examples whose average distances (a measure of similarity/dissimilarity) to the three farthest positive examples are the smallest; Method 2 involved selecting the negative examples whose average distances to the three closest positive examples are the smallest; Method 3 involved selecting the negative examples whose average distances to the three closest positive examples are the largest; Method 4 involved removing those examples that participated in Tomek links (see [74] for a definition); Method 5 involved selecting negative examples randomly. Ma concluded that random undersampling did not perform the best. ‘In general, the first and third undersampling methods work well with all feature selection methods. We have a very high recall after performing undersampling techniques. However, we have a big trade-off in precision’ ([40] p. 75).

*Aggressive undersampling* as defined by Wallace (in the context of active learning) involves discarding the majority examples (i.e., excludes) nearest the current separating hyperplane [11]. The separating hyperplane represents the border between the two classes: includes and excludes. Therefore, by throwing away those nearest to the hyperplane, we are discarding those that are the most ambiguous as to whether they should be in the include or exclude class. As such, the items that are more likely to be excludes are sent to the human reviewer for manual screening, which are then used to retrain the classifier. The logic behind this approach is to ‘explicitly push the decision boundary away from the minority class [includes], as it has been observed that when there is class imbalance, SVMs are prone to discovering hyperplanes that are closer to the minority class than the ideal separating boundary, resulting in false negatives’ ([11] p. 5).

Wallace (2010a) [11] compared naive random sampling and aggressive undersampling in their evaluation of active learning with an SVM classifier. They concluded that aggressive undersampling performed better [11]. Miwa et al. compared aggressive undersampling with a range of other options and found that whilst it outperformed the other strategies at the beginning of the active learning sequence, other methods overtook it as screening progressed [45].

It is difficult to draw conclusions across the papers, as the two that conducted a comparison differed in many other dimensions (classifier, reviews tested, etc.). This requires further exploration.

Cohen and colleagues observed that any kind of sampling strategy can result in the exclusion of a large proportion of the possible sample available from which the classifier can ‘learn’ [66]. ‘To address this, we sample the nontopic data, creating several different priming SVM models, and extract the support vectors from each of these models to use as priming vectors. The nontopic data are rejection sampled, that is, sampled without replacement. The probabilities of inclusion for each sample within a given nontopic are adjusted so that approximately the same number of samples from each nontopic is included.’ In their experiments they used 20 resamples.

#### Other methods for dealing with class imbalance

Some authors claimed that certain classifiers are particularly well suited to imbalanced datasets. Bekhuis Frunza, Kouznetsov and Matwin claimed that complement naïve Bayes (CNB) is suitable for imbalanced data, particularly when implemented in Weka [24, 30, 41, 54, 60, 61]. Frunza and colleagues compared CNB with other classifiers (decision trees, support vector machine, instance-based learning and boosting) but concluded that CNB always performed better; it is not clear, however, whether this is because of the class imbalance problem or other differences between the approaches [24, 61].

Some authors have suggested that the selection of features for text mining might be important in addressing class imbalances. Although they did not test it in their paper, Bekhuis et al. suggested that selecting features within the positive (include) and negative (exclude) classes before grid optimization, rather than across all items, would be appropriate for dealing with class imbalance [30]. Frunza explicitly compared classifiers that had been ‘boosted’ in terms of having more representative features for the included class (a balanced dataset) with typical feature selection technique (imbalanced dataset) but found no significant difference between these two approaches [24].

### Updates versus ‘new’ reviews

Out of the 44 studies, the context of 36 was a new review, eight a review update, and for two studies the review context was not the primary area of investigation (the issue was the performance of classifiers). The context of new reviews is challenging, because there is so little training material available at the start of screening on which to conduct any machine learning. Whilst the concept of obtaining an unbiased set of training material using a random sample is widely employed, Wallace and colleagues have outlined an explicit iterative method to determine whether the variation in likely ‘includes’ has been explored adequately enough for active learning to begin [11]. They do this drawing on the work of Brinker who has developed methods for incorporating diversity in active learning by evaluating the stability of a measure of similarity between ‘included’ citations between iterations [75]. Once the measure of similarity ceases to change between iterations, the sample can be considered ready to perform active learning.

In contrast, whilst the review update might appear to be the more straightforward situation, since there are preexisting citation decisions on which to ‘learn’, some of the earliest work included in our review—by Cohen—shows that review updates face many challenges of their own [35, 66, 68, 69]. In particular, the issue of ‘concept drift’ looms large over the review update. As Bekhuis points out, there are many changing variables in a review update—the team, the searches and even aspects of the question may all change—and the data from the original review may cease to be a reliable indicator of what should be included in the new one [60]. Dalal and colleagues attempted to mitigate the effects of concept drift but were not entirely successful [70].

#### Additional information on this topic

Online learning methods which treat datasets as a stream, updating their model for each instance and discarding it after updates, can be used for new reviews. Some online learning algorithms adapt their models quickly to new coming data and can be adapted to deal with slight concept drift [76]. Domain adaptation, multi-task learning and transfer learning can improve models for a specific review by using related information from other reviews and problems. Such learning methods support the learning of multiple, related review targets [77].

How has the workload reduction issue been evaluated?

The following section addresses research question 4: *How has the workload reduction issue been evaluated?* There are three aspects that we explore: what has been compared and through what research design; and what metrics were used to evaluate the performance of the technologies?

What has been compared, using what research design?

The vast majority of evaluations used a retrospective design; that is, they assessed performance against the ‘gold standard’ judgements made in a completed systematic review [11, 25, 30, 34, 36–45, 47, 48, 51, 52, 55, 56, 59–62, 66, 68, 70] (*n* = 27). In contrast, prospective designs are those in which the technology was assessed in a ‘live’ context; that is, as the review was being conducted. Seventeen studies employed a prospective design, of which five were self-described as ‘case studies’ [31, 46, 50, 57, 63], four were controlled trials [13, 14, 64, 65], and eight were other prospective designs [23, 24, 35, 49, 53, 54, 58, 69].

The type of design is important, as prospective designs have the potential to tell us more about how the text mining technologies might work when implemented in ‘real life’. Whilst retrospective simulations are essential in determining the relative performance of different classifiers or establishing the optimal parameters of a classifier, some of the difficulties of implementing such technologies in a live review cannot be taken into account adequately (e.g., reviewer over-inclusiveness at different stages of the process, which might ‘mislead’ the classifier about what an include ‘looks like’). Moreover, many of the evaluations are of relatively ‘neat’ datasets, in that they have a sufficient number of includes on which to train (even if they are the minority class). How does text mining cope when there is a tiny number of includes, or in a so-called ‘empty’ review, in which there are no included studies?^b^

Related to the issue of *how* the technologies were evaluated is the question of *what* was evaluated. Most of the evaluations conducted to date (*n* = 29) make some form of comparison between different algorithms or methods for text mining [11, 23–25, 30, 34, 36, 37, 39–43, 45, 49, 51–55, 58, 60–62, 66, 68–70]. The main issues evaluated are: the relative effectiveness of different methods for classifying studies (i.e. ‘classifiers’ and different options for using them (‘kernels’)); how different approaches to ‘feature selection’ (the way that aspects of studies—e.g. their titles, abstracts and MeSH headings are encoded for machine learning) impact on performance; how effective different approaches to separating different pieces of ‘intelligence’ about the study are (e.g. separating titles from abstracts); and whether performance differs depending on how many studies are used for the initial training. The remaining 16 evaluations do not compare aspects of the methodology; rather, they report on the effectiveness of one chosen method for implementing text mining [13, 14, 31, 35, 38, 44, 46–48, 50, 56, 57, 63–65].

Unsurprisingly, study design is associated with certain types of comparisons (see Table 3). The four controlled trials all compared human performance with machine performance but did not compare different aspects of text mining technologies. None of the five case studies compared text mining features either, with an emphasis instead on how workload could be reduced in an ongoing review. The retrospective simulation studies tended to compare more features of text mining than other prospective studies, perhaps because of the comparative ease with which adaptations to the text mining approach can be made in a retrospective evaluation.

**Table 3:** **Cross tabulation showing the number of studies employing certain research designs by the aspects of text mining that were compared (** ***n*** **= 44)**

What aspect of text mining was compared	Retrospective simulation	Prospective—case study	Prospective—controlled trial	Prospective—other	Total—what was compared
Classifiers/ algorithms	13	0	0	3	16
Number of features	2	0	0	0	2
Feature extraction/sets (e.g., BoW)	8	0	0	2	10
Views (e.g., T&A, MeSH)	5	0	0	1	6
Training set size	2	0	0	0	2
Kernels	2	0	0	0	2
Topic specific versus general training data	3	0	0	1	4
Other optimisations	9	0	0	4	13
No comparison	5	5	4	1	
Total**—**study design (duplicates removed)	(27)	(5)	(4)	(8)	

*Note.* Many studies compared more than one aspect of text mining, therefore column total for ‘Total**—**what was compared’ sums to greater than 44. The row for ‘Total**—**study design (duplicates removed)’ shows the number of studies of each design type rather than the column totals, as the column totals would include duplications of the same studies that compared multiple aspects of text mining technologies.

### Metrics for assessing classifier performance

In this section, we address *research question 3*: What metrics are available for evaluating the performance of the approaches, in terms of both effectiveness and efficiency? The metrics are presented in order from the most popular to the least in Table 1. Most studies reported more than one performance metric and generally considered the importance of both identifying relevant studies *and* reducing workload for the reviewers. The metrics are defined in Table 1.

There are various arguments used throughout the literature as to which metric is the most appropriate. It should be noted that not all metrics are suitable for all evaluation designs or text mining technology types. For instance, coverage is only suitable for active learning approaches, whilst Cohen noted that ‘If the task is not to separate documents into positive and negative groups, but instead to prioritise which documents should be reviewed first and which later, then precision, recall and F measure do not provide sufficient information’ (p. 121) [68].

Measures that allow the trade-off between recall and precision to be taken into account on a review-by-review basis seem particularly useful, as they allow reviewers to change the relative importance of these two metrics depending on priorities in a given review. These metrics include notably the F measure, work saved over sampling and utility, which are summarised below.

*F measure* is a weighted harmonic mean of precision and recall. The weighting can be determined on a review-by-review basis, allowing reviewers to assess the relative importance of recall and precision in their context.

*Work saved over sampling* (WSS) indicates how much work (in terms of number of items needed to screen) is saved over and above the work saved by simple sampling for a given level of recall. It is typical to use a recall level of 0.95. See Cohen et al. [36].

*Utility* is relevant for active learning approaches and is calculated based on yield and burden. Yield represents the fraction of includes in the data pool that are identified by a given method, and burden represents the fraction of includes in the data pool that have to be annotated/reviewed by reviewers. The formula to calculate utility includes a weighting factor so that the reviews can specify the relative importance of yield and burden. This weighting factor has been established for some contexts but might need to be re-established for application in other settings [25].

It is clear from the three metrics above that there is a subjective element to the performance metrics, as it is up to the evaluators to determine thresholds and weighting values. Whilst this has the advantage of making the metrics tailored to the review and evaluation context, it (a) makes it difficult to compare across studies that use different thresholds/weights in their calculations, and (b) it is not always transparent or justified as to how the thresholds/weights were selected.

#### Evaluation metrics that emphasise high recall

As mentioned above, many studies discussed the importance of high recall without necessarily making explicit adaptations to their text mining approach. They do, however, consider the importance of high recall in their choice of metric when evaluating the performance of the text mining technology. Examples included:

- ● Bekhuis (2012) used F3—a summary measure that overweights recall relative to precision—because they felt this was more in keeping with reviewer behaviour (than a metric which weights them equally) [59]
- ● Kouznetsov (2010) used false negatives (relevant articles mistakenly ranked at the bottom of a ranked list) as their primary performance measure [54]
- ● Wallace (2011) [58] used U19—a weighted metric in which recall is 19 times as important as cost. The value of 19 was determined through an expert consultation process [25] (see Wallace [11])
- ● Dalal (2013) evaluated performance using a range of probability thresholds to better consider the impact on observed performance of using different recall and precision trade-offs: one metric was based on ‘sensitivity-maximising thresholds’ whilst another ‘preserved good sensitivity whilst substantially reducing the error rate [false positives]’ (p. 348) [70]

In contrast to most of the studies in this review, Dalal (2013) argued that ‘neither error minimization nor sensitivity maximisation are absolute goals’ (p. 348) [70]. In fact, Fiszman and colleagues (2008, 2010) used the F0.5 measure, which weights precision more highly than recall [38, 53]. They argue that clinical practice guideline developers value precision more than recall and therefore performance should be evaluated on this basis. This suggests that the relative importance of recall and precision might vary from context-to-context, and a high recall should not be assumed to be more important than high precision (though in most systematic review guidance—and practice—maximising recall is prioritised).

#### Evaluation metrics that account for class imbalance

As with the issue of the importance of high recall in systematic reviews, some authors have reflected the class imbalance problem in their choice of evaluation measure. Cohen (2010) argued that the AUC is independent of class prevalence [24, 35], whilst Frunza [24] reported the F measure for the same reason. The choice of evaluation metric should consider whether class imbalance is likely to bias the results.

#### Further information on this topic

We should note that other evaluation metrics can also account for class imbalance. For example, if you care about both the TPs and the TNs, you’d use ROC-AUC, but if you only care about the TPs, you might prefer PR_AUC [78]. See also [79].

### Implementation challenges

The following section attempts to answer research question 5: *What challenges to implementation emerge from reviewing the evidence base?* Whilst almost all of the papers concluded that text mining was a ‘promising’ approach to reduce workload in the screening stage of a systematic review, it was not always clear how these technologies would be rolled out for use in ‘live’ reviews. A few issues became clear that need to be considered for the knowledge gained in these studies to have practical application (all of which apply to other uses of automation and semi-automation in systematic reviews [80]).

### Deployed systems

Only six different systems (reported in 12 papers) are currently ‘deployed’—that is, are in a packaged system that a reviewer could use without having to do any computer programming. Some are bespoke systematic review systems, whereas others are more generic software for predictive analytics which can be used in a systematic review. The bespoke systems for systematic reviews which were used in evaluations in this review are: Abstrackr [49, 50], EPPI-Reviewer [31, 57], GAPScreener [51] and Revis [64]. Many generic software applications support the kinds of machine learning evaluated in this review; the two that were used in our included papers were Pimiento [62] and RapidMiner [59, 60]. However, even though no programming may be required to use these tools, reviewers using the systems are likely to require some training to be able to use them. Given concerns about the need for high recall, imbalanced datasets, etc., these are not packages that can be used without understanding some of the behind-the-scenes decisions that are made with respect to handling the data.

### Replication of evaluations

Only one study in the evidence base represents a true replication of another study (Felizardo [65]). There are some partial replications that used the same dataset; notably, Cohen and colleagues and Matwin and colleagues had an ongoing correspondence in the Journal of the American Medical Informatics Association in which they presented results across the same review datasets using different classifiers and parameters. Most studies differ in many ways: datasets used, classifiers tested, feature selection processes applied, citation portions viewed, comparisons made, study designs employed, metrics used for evaluation, etc. This makes it impossible to compare results across studies directly. It also makes it difficult to conclude whether any particular aspect of the abovementioned differences is particularly important to adopt or fruitful to explore in future research.

It is hoped that future evaluations will attempt more replications of the same methodological applications but on different datasets, to determine whether findings hold when applied to new topic areas. For instance, Miwa [45] reported that a particular approach did not perform as well on ‘messy’ social science datasets as it did for ‘cleaner’ clinical datasets that had been used elsewhere (though other enhancements can make up for some of this deficit)—these sorts of partial replications of the method are helpful in understanding the cross-review and cross-disciplinary applicability of the evaluation findings [45].

### Scalability

A further concern is whether some of the approaches will work on very large datasets—that is, can they be ‘scaled up’ from the small datasets used in the evaluations to the larger datasets that are often encountered in systematic reviews. The largest evaluation was on a dataset of more than 1 million citations [31], although that was a case study (and an extreme one at that!); the second largest evaluation was on a dataset of 47,274 [24]. However, the vast majority were conducted on review datasets that were well below 5,000 items, with the smallest datasets being only 57 items (20 in the training set, 37 in the test set; [64, 65]).

Given that the purpose of using such technologies in systematic reviews is to reduce screening workload, then it seems appropriate to test them on datasets for which the workload is large or even unmanageable. Although we can extrapolate from the smaller datasets to larger reviews, there is a limit to how much we can assume that the technologies will be able to detect true positives in such large (and thereby presumably more diverse) datasets.

The issue of scalability is particularly relevant to the visual text mining approaches, as discussed earlier in the paper. Consideration will need to be paid to how to represent connections between papers visually when many items are in the dataset; the visual image could be too overwhelming to be of any use in aiding human information processing. Either adaptations to such tools will need to be made for scaling up, or an upper threshold of number of items in the dataset might need to be established.

#### Further information on this topic

Methods such as stream-based active learning are promising in handling large-scale data instances [81]. Stream active learning is closely related to online learning [3.3.4], but as it does not need to store all the instances in active learning, it can handle large-scale data instances.

### Suitability. Appropriateness of TM for a given review

This systematic review has aimed to identify all the relevant studies concerning the use of text mining for screening, finding that it is a relatively new field with many gaps in the evidence base. One significant gap is the limited range of topics and types of study within the reviews which have been used to evaluate the text mining methods. On the whole, they are concerned with identifying RCTs in clinical areas and there are almost no examples outside the health and biomedical sector apart from a discrete set in the area of software engineering. This is not surprising, since these are the areas that text mining for other purposes is most common, but it is an important area for future research, because general literature is more challenging to text mine because of the variability of concepts, text categorisation, etc.

Bekhuis and Demner-Fushman tested this explicitly in their study of 2010, looking for non-randomised, as well as randomised, controlled trials (though still in the medical domain) [59]. Their findings are promising, though they are concerned about the possibility of ‘over-fitting’ and the danger of building a classifier that does not recognise the true scope of relevant studies. They identify a specific type of SVM classifier and conclude that their method may be able to identify non-randomised studies with a high degree of recall—as long as the citations on which the machine learning can ‘train’ encapsulate the full range of the potentially relevant studies. Miwa et al. test explicitly the difference in performance of the same machine learning approaches between ‘clinical’ and ‘social science’ reviews [45]. They found that text mining performance was slightly poorer in the social scientific literature than the clinical domain and that certain enhancements could improve this.

Wallace and colleagues suggest a method to be used in review updates which enable reviewers to determine whether a semi-automated approach is viable [48]. They recommend a ‘cross-fold validation’ test, whereby the database of studies from the original review is split into parts (say, 10) and the classifier successively trained on 90% of the data, leaving 10% for assessing its performance. Performance is then averaged over the 10 iterations and if acceptable, then the use of automation for the update of that specific review can be recommended.

#### Further information on this topic

Most text mining systems used in systematic reviews use shallow information e.g. bag-of-words and their combinations, e.g., kernels. Natural language processing techniques such as syntactic parsing can be employed to engineer more discriminative features. Furthermore, unsupervised feature learning or dimensionality reduction approaches can be employed to build feature representations suitable for specific domains as well as finding queries to relieve hasty generalisations as mentioned in 3.3.2 [82].

### Over-inclusive screeners

The success of most automated approaches relies upon ‘gold standard’ training data; that is, citations that the machine can assume have been correctly designated as relevant or irrelevant. Using these data, the machine is then able to build a model to designate such classifications automatically. Usually, these gold standard training data take the form of decisions made by reviewers when screening a proportion of the studies of interest. Unfortunately, these decisions may not actually be ‘gold standard’ training data, because reviewers are trained to be over inclusive, and to retrieve the full text whenever they are in doubt—even if the most likely final decision is that it is irrelevant. Such decisions may mislead the classifier and generate a model which incorrectly classifies irrelevant studies as relevant. Bekhuis et al. acknowledge this as a potential problem, but go on to argue then that to ‘be worthwhile, a classifier must return performance better than this baseline to ensure reduced labor’ [60]: a pragmatic way of looking at how machine learning might potentially assist in systematic reviews. Frunza et al. also encountered this challenge, finding that the best way of mitigating the effects of reviewer over-inclusivity was to base the machine learning on designations that were the result of two reviewers’ opinions—after disagreements had been resolved [61]. This solution is clearly only possible when two reviewers are reviewing every abstract—something which is common, but by no means universal, practice.

#### Further information on this topic

A machine learning-based method able to deal with over-inclusive screening as well as data imbalance is cost-sensitive learning [83]. Cost-sensitive learning assigns misclassification costs to certain types in learning and adapts machine-learning methods for task-specific criteria. It is as competitive as or better than sampling methods for unbalanced datasets [84], and it is also employed in active learning [85].

## Discussion

### Summary of key findings

This review asked five research questions, which we have addressed through synthesising the evidence from 44 evaluations of the use of text mining for reducing screening workload in systematic reviews.

The *first research question* related to the state of the evidence base, which we conclude to be both active and diverse. The timeline indicates that the field is evolving rapidly, with new issues being tackled almost every year since its application to systematic reviews. However, this also hints at an issue that was elaborated on throughout this paper—that is, there is almost no replication between studies or collaboration between research teams, making it difficult to establish any overall conclusions about best approaches.

The *second research question* related to the purpose of using text mining to reduce workload and the methods used for each purpose. For reducing the number needed to be screened, it is reasonable to assume that the more interactive approach offered by a ranking or prioritisation system and the active learning approaches will have greater user appeal than a strict classifier approach in ‘new’ reviews (as opposed to review updates). This is because reviewers might be uncomfortable with handing over too much control to an automated system. Also, when using a ranking or prioritisation approach, reviewers are able to search more sensitively than is currently the norm and screen the same number of studies as they currently would; the effort spent screening manually would thus be focused on those studies identified as being the *most relevant* retrieved in the search, enabling these reviews to identify more relevant studies than is currently the case.

For using text mining to replace a second human screener, classifiers were used to make explicit in/out decisions and those decisions were compared with a human reviewer. This approach is likely to have strong appeal amongst the systematic review community because, whilst it reduces the resources required to screen items, 100% of the items identified through searching are still viewed by a human screener. This could combat concerns about false negatives assigned by an automated screener. A further potential benefit of such a system is that it ‘could deliver quality assurance both by confirming concordant decisions and by naming studies associated with discordant decisions for further consideration’ (Bekhuis [60], p. 9) (One possible weakness of this approach is that it necessarily assumes that any mistakes made by the human screener are essentially at random, and not because of some systematic misapplication of the inclusion criteria, which might be picked up and addressed if two reviewers were working in tandem.).

Reducing workload by increasing the rate (or speed) of screening was a little researched topic, exclusively limited to the visual data mining approach and largely championed by one research group. A major limitation of these evaluations—and potentially for the wider applicability of these approaches—is that the approach has only been tested on very small datasets. The largest dataset consisted of only 261 items to be screened [13]. It is unclear whether such an approach could be scaled up to be applied in other disciplines in which thousands of items might need to be screened, though the authors argue that upscaling is indeed possible. The efficient citation assignment approach evaluated by Wallace et al. [49] may also be promising for larger reviews where the expertise of the reviewers is known.

Improving workflow efficiency through screening prioritisation is likely to appeal to systematic reviewers as it allows for reviewers to screen 100% of the titles and abstract but with a range of benefits. Benefits discussed in the literature included: understanding the inclusion criteria sooner, getting up to speed on new developments in review updates, starting full-text document retrieval sooner and starting the data extraction and synthesis processes in parallel with screening the ‘tail end’ of the list of items (in which there are expected to be very few or zero relevant items).

The *third research question* related to the contextual problems of applying text mining to systematic review screening and how they have been addressed in the literature. We found various attempts to address the importance of high recall for systematic reviews (vote counting; specialist algorithms; and human input). Whilst all evaluations reported good recall, the studies used different adaptations; so it is impossible to conclude whether any approach is better than another—and in which context. However, human input is likely to have intuitive appeal to systematic reviewers, as it allows for a human sense-check of the terminology preferences determined by the machine.

One important distinction to make when evaluating the utility of machine learning in screening is whether one is creating a new review or updating and existing one. Given the existence of the preexisting data for review updates, it is often possible to know in advance the likely performance of using text mining, enabling reviewers to make an informed decision about its potential in that specific review. Such a situation does not pertain in new reviews, and the risk of hasty generalisation is a ‘known unknown’ here, as are the risks and benefits of adopting a semi-automated approach.

The lack of replication and testing outside the biomedical sphere makes it difficult to draw conclusions about the general effectiveness of these technologies. Certainly, where technical jargon is utilised, most approaches appear to offer efficiency savings; and in the few instances of their application outside the medical domain they again can be effective, though potentially slightly less so.

The *fourth research question* considered how the workload reduction issue has been evaluated. Here, it was impossible to synthesise study findings quantitatively, because each used different technologies in (usually) different reviews. On the whole, most suggested that a saving in workload of between 30% and 70% might be possible (with some a little higher or a little lower than this), though sometimes the saving in workload is accompanied by the loss of 5% of relevant studies (i.e. a 95% recall).

The *fifth research question* considered the challenges to implementation that emerged from reviewing the evidence base. Here, we found few deployed systems, which limits the ability of reviewers to try out these technologies, but also, given the limitations in the evidence base identified above, there is probably a need for specialist advice whenever they are used in a live review—and certainly if workload reduction is planned (i.e. if their use extends beyond prioritising screening). We also found a lack of replication studies, which makes it difficult to compare the efficacy of different approaches across review contexts, and few evaluations outside the biomedical domain. Challenges in using such technologies include questions about how they might scale to large reviews and how to model accurate classifiers when the decisions made by reviewers are likely to err on the side of caution, and hence be over-inclusive.

### Strengths and limitations of this review

To the best of our knowledge, this is the first systematic review that has brought together evidence concerning the use of text mining for screening in systematic reviews. We have identified a varied, innovative and potentially extremely important evidence base—which one day may do much to improve review efficiency and so improve decision-making. We hope that this review will help the different areas of the field to ‘speak’ to one another and so facilitate the development of the field as a whole.

As there are no other systematic reviews of this area, we had a broad review question, which encompassed any approach. This has enabled us to identify the cross-cutting issues in the field but has limited the quantity of technical information that we have been able to present. For example, a narrower review focused solely on active learning might be able to delve into the specifics in more detail.

An inevitable limitation due to setting the scope of the review to evaluations of text mining approaches *within* systematic reviews is that relevant research in other areas is excluded. For example, if we had reviewed all potentially relevant research about text mining and active learning (an almost impossible task!), other technologies and approaches, beyond those so far evaluated in systematic reviews, might well have come to light. Whilst this limitation was impossible to avoid, it is nevertheless a significant limitation, because only a small subset of possible approaches to, for example, feature selection/enrichment and distance analytics, have been tested within the systematic review literature. The field of text mining contains many more possibilities—and some may be more effective and appropriate than those so far evaluated.

A limitation which applies to any systematic review is that we may not have managed to find *every* relevant study. This was highlighted to us during the peer review process when another relevant study came to light. This study was focused on a text mining approach and utilised data from systematic reviews as its test scenario [71]. There may be other papers like this one which we have inadvertently missed.

### Further possibilities

It is interesting to note that text mining approaches to support screening have followed the human reviewer’s initial approach of using titles, abstracts and keywords. The human reviewer will retrieve full text for further review, but typically text mining approaches so far have not processed full text in support of the screening process. There are essentially three issues to consider here. Firstly, there is the issue of how well a title, abstract and metadata can satisfy a complex information need. For example, regarding use of an abstract to determine what claims are being made, Blake found that, in biomedicine, fewer than 8% of the scientific claims made in full-text articles were to be found in their abstracts, which would certainly motivate the need to process full text [86].

Cohen and colleagues have investigated more widely the implications for text mining of processing abstracts as opposed to full-text articles, and moreover mention a second issue, to do with problems that may arise for systems in going from the processing of abstracts to the processing of full text, but note that there are opportunities to be exploited in so doing [87]. Text mining technology has, however, improved greatly since that publication. There are now text mining systems that process large amounts of full text and that support sophisticated semantic search. For example, Europe PubMed Central, a large archive for the Life Sciences, showcases on its Labs site a semantic search system, EvidenceFinder, that is underpinned by deep parsing, conducted in a cloud environment, of some 2.5 m articles to yield over 83 m searchable facts (http://labs.europepmc.org/evf).

Text mining can increasingly handle deep analysis of full-text context, at scale, thus it would be natural to move towards exploiting such a capability in support of systematic reviews. However, this leads into the third issue, concerning copyright, licencing and lawful access to full-text content for text mining purposes. Reviewers already run into this issue when they find that their institution does not subscribe to some journal, for example. However, even if one’s institution does have the relevant subscription, licencing terms may explicitly disallow text mining or allow it but place constraints on use of its results. This is a hot topic, with researchers claiming that ‘the right to read is the right to mine’ (Open Knowledge Foundation). Open Access publications are not subject to the same constraints as subscription-based content; however, there is growing concern amongst researchers and funding bodies that opportunities are being lost to advance knowledge and boost innovation and growth due to restrictive copyright and licencing regimes that are unsuited to the digital age [88, 89]. Most recently, the UK has passed legislation to legalise text mining for non-commercial use (http://www.legislation.gov.uk/uksi/2014/1372/regulation/3/made). There is thus a valuable opportunity for the systematic reviewing community in the UK at least to work closely with its text mining community to exploit the benefits of full-text processing, particularly to improve screening and to reduce the need for humans to laboriously move from abstract to full text to carry out a more specific check for relevance.

The use of automation to assist in study selection is possibly the most advanced of all the areas where automation in systematic reviews is being developed; but others range from writing sections of the report, formulating the review question and automated data extraction and quality assessment [90–93].

### Recommendations

#### Recommendations for research

- ● More replications using the same text mining methods on different datasets are required.
- ● Likewise, different methods using the same dataset are also needed in order genuinely to compare one with another.
- ● To facilitate the above, data on which evaluations are based should be made public as often as possible.
- ● The testing of the methods reviewed here in other disciplines is urgently required. For example, the field of Development Studies may be more complex and thus demand more of the text mining (promoting more innovation to overcome new hurdles).

#### Recommendations for reviewing practice

- ● Reviewers should engage with the computer science community to develop and evaluate methods and systems jointly.
- ● Using text mining to prioritise the order in which items are screened should be considered safe and ready for use in ‘live’ reviews.
- ● The use of text mining as a ‘second screener’ may be used cautiously in the knowledge that the assumption is that the human reviewer is not missing relevant studies systematically.
- ● The use of text mining to eliminate studies automatically should be considered promising, but not yet fully proven. In highly technical/clinical areas, it may be used with a high degree of confidence; but more developmental and evaluative work is needed in other disciplines.

## Conclusion

Whilst there is a relatively abundant and active evidence base evaluating the use of text mining for reducing workload in screening for systematic reviews, it is a diverse and complex literature. The vast array of different issues explored makes it difficult to draw any conclusions about the most effective approach. There are, however, key messages regarding the complexity of applying text mining to the systematic review context and the challenges that implementing such technologies in this area will encounter. Future research will particularly need to address: the issue of replication of evaluations; the suitability of the technologies for use across a range of subject-matter areas; and the usability and acceptability of using these technologies amongst systematic review (non-computer scientist) audiences.

## Endnotes

^a^A ‘method’, in the context of this review, is the application of a specific technology or a process within a systematic review. This is a somewhat broad definition which includes, for example, both the use of a classifier to classify citations as being relevant/irrelevant; and also the ‘active learning’ approach, which incorporates a classifier as part of its process. This broad definition reflects the practical purpose of this review—we are interested in approaches that can be applied in systematic reviews, and these may be individual tools, combinations of tools or processes for using them.

^b^The practicalities of implementing text mining in live reviews are the subject of a current project by the EPPI-Centre and NaCTeM, which aims to address some of these issues. Project URL: http://www.ioe.ac.uk/research/63969.html.

## Electronic supplementary material

Additional file 1: **Appendix A.** Search strategy. **Appendix B.** Data extraction tool. **Appendix C.** List of studies included in the review (*n* = 44). **Appendix D.** Characteristics of included studies. (DOC 151 KB)

Additional file 2: **Flow diagram.** (DOC 58 KB)

## Abbreviations

CNB: complement naïve Bayes
FNLR: false negative learning rate
HTA: health technology assessment
LISTA: Library, Information Science & Technology Abstracts
NLP: natural language processing
SVM: support vector machine
VDM: visual data mining
WSS: work saved over sampling.
