# Erratum to: Using text mining for study identification in systematic reviews: a systematic review of current approaches

**DOI:** 10.1186/s13643-015-0031-5

**Published:** 2015-04-28

**Authors:** Alison O’Mara-Eves, James Thomas, John McNaught, Makoto Miwa, Sophia Ananiadou

**Affiliations:** Evidence for Policy and Practice Information and Coordinating EPPI-Centre, Social Science Research Unit, Institute of Education, University of London, London, UK; The National Centre for Text Mining and School of Computer Science, Manchester Institute of Biotechnology, University of Manchester, 131 Princess Street, Manchester, M1 7DN UK; Toyota Technological Institute, 2-12-1 Hisakata, Tempaku-ku, Nagoya, 468-8511 Japan

## Erratum

Following publication of our article [1], it has come to our attention that two of the formulae in Table 1 were incorrect. The formulae for the measures of precision and burden have been corrected (Table 1). We are publishing this erratum to update these formulae to the following:

**Table 1:** **Definitions of performance measures reported in the studies**

**Measure**	**#**	**Definition**	**Formula**
***Recall (sensitivity)***	22	Proportion of correctly identified positives amongst all *real* positives	$\frac{\mathrm{TP}}{\mathrm{TP}+\mathrm{F}\mathrm{N}}$
***Precision***	18	Proportion of correctly identified positives amongst all positives.	$\frac{TP}{TP+FP}$
***F measure***	10	Combines precision and recall. Values of *β* < 1.0 indicate precision is more important than recall, whilst values of *β* > 1.0 indicate recall is more important than precision	${F}_{\beta, k}\kern0.5em =\kern0.5em \frac{\left({\beta}^2+1\right){\mathrm{TP}}_k}{\left({\beta}^2+1\right){\mathrm{TP}}_k+{\mathrm{FP}}_k+{\beta}^2{\mathrm{FN}}_k}$ Where *β* is a value that specifies the relative importance of recall and precision.
***ROC (AUC)***	10	Area under the curve traced out by graphing the true positive rate against the false positive rate. 1.0 is a perfect score and 0.50 is equivalent to a random ordering	
***Accuracy***	8	Proportion of agreements to total number of documents.	$\frac{\mathrm{TP}+\mathrm{T}\mathrm{N}}{\mathrm{TP}+\mathrm{F}\mathrm{P}+\mathrm{F}\mathrm{N}+\mathrm{T}\mathrm{N}}$
***Work saved over sampling***	8	The percentage of papers that the reviewers do not have to read because they have been screened out by the classifier	$\mathrm{W}\mathrm{S}\mathrm{S}\ \mathrm{at}\ 95\%\ \mathrm{recall} = \kern0.5em \frac{\mathrm{TN}+\mathrm{F}\mathrm{N}}{N-0.05}$
***Time***	7	Time taken to screen (usually in minutes)	
***Burden***	4	The fraction of the total number of items that a human must screen (active learning)	$Burden=\frac{t{p}^T+t{n}^T+f{p}^T+t{p}^U+f{p}^U}{N}$
***Yield***	3	The fraction of items that are identified by a given screening approach (active learning)	$\mathrm{Yield}\kern0.5em =\kern0.5em \frac{{\mathrm{tp}}^T+{\mathrm{tp}}^U}{{\mathrm{tp}}^T+{\mathrm{tp}}^U+{\mathrm{fn}}^U}$
***Utility***	5	Relative measure of burden and yield that takes into account reviewer preferences for weighting these two concepts (active learning)	$\frac{\beta \cdot \mathrm{yield}+\left(1\kern0.5em -\kern0.5em \mathrm{burden}\right)}{\beta +1}$ Where β is the user-defined weight
***Baseline inclusion rate***	2	The proportion of includes in a random sample of items before prioritisation or classification takes place. The number to be screened is determined using a power calculation	$\frac{n_i}{n_t}$ Where *n*_*i*_ = number of items included in the random sample; *n*_*t*_ = total number of items in the random sample
***Performance (efficiency)*** ^***a***^	2	Number of relevant items selected divided by the time spent screening, where relevant items were those marked as included by two or more people	$\frac{\mathrm{Selected},\kern0.5em \mathrm{relevant}\kern0.5em \mathrm{items}}{\mathrm{Time}}$
***Specificity***	2	The proportion of correctly identified negatives (excludes) out of the total number of negatives	$\frac{\mathrm{TN}}{\mathrm{TN}+\mathrm{F}\mathrm{P}}$
***True positives***	2	The number of correctly identified positives (includes)	TP
***False negatives***	1	The number of incorrectly identified negatives (excludes)	FN
***Coverage***	1	The ratio of positives in the data pool that are annotated during active learning	$\frac{{\mathrm{TP}}^L}{{\mathrm{TP}}^L+{\mathrm{FN}}^L+{\mathrm{TP}}^U+{\mathrm{FN}}^U}$ Where *L* refers to labelled items and *U* refers to unlabelled items
***Unit cost***	1	Expected time to label an item multiplied by the unit cost of the labeler (salary per unit of time), as calculated from their (known or estimated) salary	time_expected_ × cost_unit_
***Classification error***	1	Proportion of disagreements to total number of documents	100 % − accuracy %
***Error***	1	Total number of falsely classified items divided by the total number of items	$\frac{\sum \left(\mathrm{F}\mathrm{P}+\mathrm{F}\mathrm{N}\right)}{\sum \left(\mathrm{T}\mathrm{P}+\mathrm{F}\mathrm{P}+\mathrm{F}\mathrm{N}+\mathrm{T}\mathrm{N}\right)}$
***Absolute screening reduction***	1	Number of items excluded by the classifier that do not need to be manually screened	TN + FN
***Prioritised inclusion rate***	1	The proportion of includes out of the total number screened, after prioritisation or classification takes place	$\frac{n_{\mathrm{ip}}}{n_{\mathrm{tp}}}$ Where n_ip_ = number of items included in prioritised sample; n_tp_ = total number of items in the prioritised sample

Precision = $$ \frac{TP}{TP+FP} $$

Burden = $$ \frac{t{p}^T+t{n}^T+f{p}^T+t{p}^U+f{p}^U}{N} $$
