# Supplementary material for: Using text mining for study identification in systematic reviews: a systematic review of current approaches
Source: Syst Rev. 2015 Jan 14;4(1):5. doi: 10.1186/2046-4053-4-5 (PMC4320539; doi:10.1186/2046-4053-4-5)
Supplement: Supplementary file 1 — Additional file 1: Appendix A. Search strategy. Appendix B. Data extraction tool. Appendix C. List of studies included in the review (n = 44). Appendix D. Characteristics of included studies. (DOC 151 KB) [file 13643_2014_321_MOESM1_ESM.doc]

# Appendix A: search strategy

The following databases were searched:

1. Library, Information Science & Technology Abstracts (LISTA) via EBSCO Host
2. Medline via ProQuest
3. LISA via ProQuest
4. Technology Research Database via ProQuest
5. Science Citation Index
6. Social Sciences Citation Index via Web of Knowledge
7. ZETOC
8. OPENgrey
9. AHRQ database of methods
10. IEEE
11. JISC
12. Cochrane Methodology Register
13. HTAIvortal <http://vortal.htai.org/?q=about/sure-info>
14. Google Scholar

Other online sources searched were:

1. NacTEM website
2. Research Synthesis Methods TOC
3. PLOS text mining collection: <http://www.ploscollections.org/article/browseIssue.action?issue=info:doi/10.1371/issue.pcol.v01.i14>
4. MillionShort.com
5. ACM digital library

The search syntax used in the database searches was tested in LISTA. We used a sensitive search strategy in the title, abstract, and keyword (where available) fields. The syntax consisted of two clusters of terms: one relating to text mining and one relating to systematic reviews:

(“text mining” OR “literature mining” OR “machine learning” OR “machine-learning” OR “automation” or “semi-automation” Or “semi-automated” OR “automated” OR “automating” OR “text classification” OR “text classifier” OR “text categorization” OR “text categorizer” OR “classify* text” OR “category* text” OR “support vector machine” or SVM OR “Natural Language Processing” OR “active learning” OR “text clusters” or “text clustering” OR “clustering tool” OR “text analysis” OR “textual analysis” OR “data mining” OR “term recognition” OR “word frequency analysis”)

AND (“systematic review*” OR “article retrieval” “document retrieval” OR “citation retrieval” OR “retrieval task” OR “identify* articles” OR “identify* citations” OR “identify* documents” OR “citation screening” OR “document screening” OR “article screening” OR “citation management” or “review management” or “evidence synthesis” or “research synthesis” OR “evidence review” OR “research review” OR “comprehensive review” or “reference scanning”)

# Appendix B - Data extraction tool

1. Evaluation context (details of review datasets tested)
   1. Review topic/s (add details within discipline)
      1. Medicine
      2. Social Sciences
      3. Software engineering
      4. Information systems
      5. Other
   2. Type of review
      1. 'New' reviews
      2. Updates
   3. Number of reviews tested on
   4. Size of reviews (add training and test set size if available)
2. Evaluation of feature selection
   *Add "not compared" to info box if not evaluated. Otherwise, specify/ code:*
   *1. What was the problem*
   *2. How was it addressed/tested*
   *3. What they found*
   1. Feature extraction approach (feature sets; document representation)
      1. Bag-of-words
         *each word is represented as a separate variable having numeric weight. The most popular weighting schema is tfidf*
      2. N-grams
      3. Second Order Co-Occurrence or Second Order Soft Co-Occurrence (SOSCO)
      4. Additional reviewer specified terms
      5. Vector Space
      6. LDA
      7. Other
      8. Unclear/ Not stated
   2. Feature content (citation portions)
      1. Titles
      2. Abstracts
      3. Subject headings (e.g., MeSH)
      4. MEDLINE (or other) index: publication type
      5. References
      6. Full citations with metadata
         *The metadata can include publication date, language, author information, MeSH headings associated with the article at the time of its publication, publication type and venue, and PMID.*
      7. LDA
      8. Human labelled features
      9. Other
      10. Unclear
   3. (Pre-)Processing of text/ features
      1. Yes - describe
      2. No (explicitly stated)
      3. Unclear/ not mentioned
3. Evaluation of classifier
   *Add "not compared" to info box if not evaluated. Otherwise, specify/ code:*
   *1. What was the problem*
   *2. How was it addressed/tested*
   *3. What they found*
   1. Type of classifier (add details)
      1. SVM
         *"SVM is based on statistical learning theory that tries to find a hyperplane to best separate two or multiple classes" Chen et al. 2005 p. 8*
      2. EvoSVM (evolutionary support vector machine)
      3. Naive Bayes
         *"assumes that all features are mutually independent within each class" Chen et al. 2005 p. 8*
      4. Complement naive Bayes
      5. k-nearest neighbour
      6. Regression based
      7. Semantic model
      8. Visual data (or text) mining (VDM or VTM)
      9. Bayesian
         *"A Bayesian model stores the probability of each class, the probability of each feature, and the probability of each feature given each class, based on the*
         *training data. When a new instance is encountered, it can be classified according to these probabilities" Chen et al. 2005 p. 8*
      10. Neural networks
          *"Based on training examples, learning algorithms can be used to adjust the connection weights in the network such that it can predict or classify unknown examples correctly. Activation algorithms over the nodes can then be used to retrieve concepts and knowledge from the network" Chen et al. 2005 p. 9*
      11. Symbolic learning and rule induction
      12. Other (specify)
   2. Kernel
      1. Linear
      2. Radial / Radial Basis Function (RBF)
      3. Polynomial
      4. Sigmoid
      5. Epanechnikov Degree 3
      6. Epanechnikov Degree 4
      7. Unclear or not relevant
   3. Method of dealing with class imbalance
      1. Weighting
      2. Undersampling (random)
      3. Undersampling (aggressive 1)
         *Instances furthest from the hyperplane are chosen (i.e. those nearby are discarded)*
      4. Undersampling (aggressive 2)
         *Instances closest to the hyperplane are chosen*
      5. Other
      6. Not specified
   4. Compared (initial) training set size
   5. Compared training data used
      *Refers to the specific articles chosen to train the classifier*
   6. Importance of high recall in SRs
   7. Method of dealing with selection bias problem
      1. Covariate shift method
4. Evaluation of active learning
   *Add "not compared" to info box if not evaluated. Otherwise, specify/ code:*
   *1. What was the problem*
   *2. How was it addressed/tested*
   *3. What they found*
   1. Method for selecting citations to be labelled
      1. Certainty
      2. Uncertainty
      3. Labelled features
      4. Meta-cognitive MEAL
      5. Predicted labelling times
      6. Proactive learning
      7. Query by committee
      8. Round-robin
      9. Other
      10. Not specified
   2. Method of dealing with hasty generalisation
      1. Reviewer domain knowledge
      2. Patient active learning
      3. Voting (ensemble classifiers)
      4. Not specified
   3. Addressed concept drift/ overinclusive screening
   4. Compared frequency of re-training
   5. Compared trigger for retraining
      *E.g., retrain every N includes versus retrain every N screened*
   6. Mark if not active learning
5. Implementation issues
   *Add "not compared" to info box if not evaluated. Otherwise, specify/ code:*
   *1. What was the problem*
   *2. How was it addressed/tested*
   *3. What they found*
   1. Is this a deployed system for reviewers to use?
      1. Yes (specify software/ platform)
      2. No
   2. Response of reviewers to using the system
   3. Appropriateness of TM for a given review
   4. Reducing number of manually labelled examples to form training set
   5. Humans give 'benefit of the doubt' = noise
6. About the evaluation
   1. Evaluation methodology
      1. Cross-validation (specify type)
         *"a data set is randomly divided into a number of subsets of roughly equal size. Ten-fold cross validation, in which the data set is divided into 10 subsets, is most commonly used. The system is trained and tested for 10 iterations. In each iteration, 9 subsets of data are used as training data and the remaining set is used as testing data. In rotation, each subset of data serves as the testing set in exactly one iteration. The accuracy of the system is the average accuracy over the 10 iterations." Chen et al. 2005, p. 11-12*
      2. Hold-out sampling
         *"data are divided into a training set and a testing set. Usually 2/3 of the data are assigned to the training set and 1/3 to the testing set. After the system is trained by the training set data, the system predicts the output value of each*
         *instance in the testing set. These values are then compared with the real output values to determine accuracy" Chen et al. 2005 p. 11*
      3. Leave-one-out
         *"Leave-one-out is the extreme case of cross-validation, where the original data are split into n*
         *subsets, where n is the size of the original data. The system is trained and tested for n iterations, in each of which n–1 instances are used for training and the remaining instance is used for testing." Chen et al. 2005 p. 12*
      4. Bootstrap sampling
         *"n independent random samples are taken from the original data set of size n. Because the samples are taken with replacement, the number of unique instances will be less than n. These samples are then used as the training set for the learning system, and the*
         *remaining data that have not been sampled are used to test the system" Chen et al. 2005 p. 12*
      5. Other
      6. Unclear
   2. Metrics used
      1. Recall
      2. Precision
      3. F-measure (specify weighting)
      4. ROC (AUC)
      5. Accuracy
      6. Coverage
         *indicates the ratio of positive instances in the data pool that are annotated during active learning.*
      7. Burden
      8. Yield
      9. Cost
      10. Utility
      11. Work saved (incl. WSS)
      12. RMSE
      13. Performance/efficiency
      14. Time
      15. True positives
      16. False negatives
      17. Specificity = TN/(TN+FP)
      18. Baseline inclusion rate
      19. Other
      20. None?
   3. Aims of evaluation
   4. What was compared?
      1. Classifiers/ algorithms
      2. Number of features
      3. Feature extraction/sets (e.g., BoW)
      4. Views (e.g., T&A, MeSH)
      5. Training set size
      6. Kernels
      7. Topic specific vs general training data
      8. Other optimisations
      9. No comparison
7. Study type descriptors
   1. Retrospective simulation (used completed review)
   2. Prospective
      *Either: text mining occurs as human screens*
      *Or: a new dataset was used as the test set*
   3. 'Case study'
   4. Controlled trial (two human groups)
8. Critical appraisal
   1. Sampling of test cases
      *Consider: cross-disciplinary, difficulty of concepts/ terminology, size of reviews. This will help to address the issue of generaliability: How generalisable is the sample of reviews selected?*
      1. Broad sample of reviews
         *e.g., clinical AND social science topics*
      2. Narrow sample of reviews
         *e.g., only drug trials*
      3. Unclear
   2. Is the method sufficiently described to be replicated?
      *Especially consider feature selection, as this is often poorly described*
      1. Yes
      2. No
9. Comments and conclusions
   1. Reviewers' comments
   2. Authors' comments not captured above
      *E.g., other limitations, interesting future directions, etc.*
   3. Overall conclusions (stated by authors)
10. Document type
    1. Journal article
    2. Conference paper
    3. Thesis
    4. Working paper or in press
    5. Article in periodical
11. Workload reduction problem
    1. Reducing number needed to screen
    2. Text mining as a second screener
    3. Increasing the rate of screening (speed)
    4. Workflow 1 (screening prioritisation)
    5. Workflow 2 (importance of high recall)
    6. Workflow 3 (scheduling updates)

# Appendix C - List of studies included in the review (n = 44)

Bekhuis T, Demner-Fushman D: **Towards automating the initial screening phase of a systematic review**. *Studies in Health Technology and Informatics* 2010, **160**(1):146-150.

Bekhuis T, Demner-Fushman D: **Screening nonrandomized studies for medical systematic reviews: a comparative study of classifiers**. *Artificial intelligence in medicine* 2012, **55**(3):197-207.

Bekhuis T, Tseytlin E, Mitchell K, Demner-Fushman D: **Feature Engineering and a Proposed Decision-Support System for Systematic Reviewers of Medical Evidence**. *PLoS ONE* 2014, **9**(1):e86277.

Choi S, Ryu B, Yoo S, Choi J: **Combining relevancy and methodological quality into a single ranking for evidence-based medicine**. *Information Sciences* 2012, **214**:76-90.

Cohen A: **An effective general purpose approach for automated biomedical document classification**. In: *AMIA Annual Symposium Proceedings.* vol. 13. Washington, DC: American Medical Informatics Association; 2006: 206-219.

Cohen A: **Optimizing feature representation for automated systematic review work prioritization**. In: *AMIA Annual Symposium Proceedings: 2008 2008*; 2008: 121-125.

Cohen A: **Performance of support-vector-machine-based classification on 15 systematic review topics evaluated with the WSS@95 measure**. *Journal of the American Medical Informatics Association* 2011, **18**:104-104.

Cohen A, Ambert K, McDonagh M: **Cross-Topic Learning for Work Prioritization in Systematic Review Creation and Update**. *J Am Med Inform Assoc* 2009, **16**:690-704.

Cohen A, Ambert K, McDonagh M: **A Prospective Evaluation of an Automated Classification System to Support Evidence-based Medicine and Systematic Review**. In: *AMIA Annual Symposium: 2010 2010*; 2010: 121-125.

Cohen A, Ambert K, McDonagh M: **Studying the potential impact of automated document classification on scheduling a systematic review update**. *BMC Medical Informatics and Decision Making* 2012, **12**(1):33.

Cohen A, Hersh W, Peterson K, Yen P-Y: **Reducing Workload in Systematic Review Preparation Using Automated Citation Classification**. *Journal of the American Medical Informatics Association* 2006, **13**(2):206-219.

Dalal S, Shekelle P, Hempel S, Newberry S, Motala A, Shetty K: **A pilot study using machine learning and domain knowledge to facilitate comparative effectiveness review updating**. *Medical Decision Making* 2013, **33**(3):343-355.

Felizardo K, Andery G, Paulovich F, Minghim R, Maldonado J: **A visual analysis approach to validate the selection review of primary studies in systematic reviews**. *Information and Software Technology* 2012, **54**(10):1079-1091.

Felizardo K, Maldonado J, Minghim R, MacDonell S, Mendes E: **An extension of the systematic literature review process with visual text mining: a case study on software engineering**. In*.*; Unpublished: 16.

Felizardo K, Salleh N, Martins R, Mendes E, MacDonell S, Maldonado J: **Using Visual Text Mining to Support the Study Selection Activity in Systematic Literature Reviews**. In: *Empirical Software Engineering and Measurement (ESEM), 2011 International Symposium on: 2011 2011; Banff*; 2011: 77-86.

Felizardo R, Souza S, Maldonado J: **The Use of Visual Text Mining to Support the Study Selection Activity in Systematic Literature Reviews: A Replication Study**. In: *Replication in Empirical Software Engineering Research (RESER), 2013 3rd International Workshop on: 2013 2013; Baltimore*; 2013: 91-100.

Fiszman M, Bray BE, Shina D, Kilicoglu H, Bennett GC, Bodenreider O, Rindflesch TC: **Combining Relevance Assignment with Quality of the Evidence to Support Guideline Development**. *Studies in Health Technology and Informatics* 2010, **160**(1):709-713.

Fiszman M, Ortiz E, Bray BE, Rindflesch TC: **Semantic Processing to Support Clinical Guideline Development**. In: *AMIA 2008 Symposium Proceedings: 2008 2008*; 2008: 187-191.

Frunza O, Inkpen D, Matwin S: **Building systematic reviews using automatic text classification techniques**. In: *Proceedings of the 23rd International Conference on Computational Linguistics: Posters: 2010 2010; Beijing China*: Association for Computational Linguistics; 2010: 303-311.

Frunza O, Inkpen D, Matwin S, Klement W, O'Blenis P: **Exploiting the systematic review protocol for classification of medical abstracts**. *Artificial intelligence in medicine* 2011, **51**(1):17-25.

García Adevaa J, Pikatza-Atxa J, Ubeda-Carrillo M, Ansuategi-Zengotitabengoa E: **Automatic text classification to support systematic reviews in medicine**. *Expert Systems with Applications* 2014, **41**(4):1498–1508.

Jonnalagadda S, Petitti D: **A new iterative method to reduce workload in systematic review process**. *International Journal of Computational Biology and Drug Design* 2013, **6**(1-2):5-17.

Kim S, Choi J: **Improving the performance of text categorization models used for the selection of high quality articles**. *Healthcare informatics research* 2012, **18**(1):18-28.

Kouznetsov A, Japkowicz N: **Using Classifier Performance Visualization to Improve Collective Ranking Techniques for Biomedical Abstracts Classification**. In: *Advances in Artificial Intelligence, Proceedings: 2010 2010; Berlin*: Springer-Verlag Berlin; 2010: 299-303.

Kouznetsov A, Matwin S, Inkpen D, Razavi A, Frunza O, Sehatkar M, Seaward L, O'Blenis P: **Classifying Biomedical Abstracts Using Committees of Classifiers and Collective Ranking Techniques**. In: *Advances in Artificial Intelligence, Proceedings: 2009 2009; Berlin*: Springer-Verlag Berlin; 2009: 224-228.

Ma Y: **Text classification on imbalanced data: Application to Systematic Reviews Automation**. Ottawa Canada; 2007.

Malheiros V, Hohn E, Pinho R, Mendonca M: **A visual text mining approach for systematic reviews**. In: *Empirical Software Engineering and Measurement, 2007 ESEM 2007 First International Symposium on: 2007 2007*: IEEE; 2007: 245-254.

Martinez D, Karimi S, Cavedon L, Baldwin T: **Facilitating biomedical systematic reviews using ranked text retrieval and classification**. In: *Proceedings of the 13th Australasian Document Computing Symposium: 2008 2008; Hobart Australia*; 2008: 53.

Matwin S, Kouznetsov A, Inkpen D, Frunza O, O'Blenis P: **A new algorithm for reducing the workload of experts in performing systematic reviews**. *Journal of the American Medical Informatics Association* 2010, **17**(4):446-453.

Matwin S, Kouznetsov A, Inkpen D, Frunza O, O'Blenis P: **Performance of SVM and Bayesian classifiers on the systematic review classification task**. *Journal of the American Medical Informatics Association* 2011, **18**:104-105.

Matwin S, Sazonova V: **Correspondence**. *Journal of the American Medical Informatics Association* 2012, **19**:917-917.

Miwa M, Thomas J, O’Mara-Eves A, Ananiadou S: **Reducing systematic review workload through certainty-based screening**. *Journal of Biomedical Informatics* 2014.

Razavi A, Matwin S, Inkpen D, Kouznetsov A: **Parameterized Contrast in Second Order Soft Co-Occurrences: A Novel Text Representation Technique in Text Mining and Knowledge Extraction**. In: *2009 Ieee International Conference on Data Mining Workshops: 2009 2009; New York*: Ieee; 2009: 471-476.

Shemilt I, Simon A, Hollands G, Marteau T, Ogilvie D, O'Mara-Eves A, Kelly M, Thomas J: **Pinpointing needles in giant haystacks: use of text mining to reduce impractical screening workload in extremely large scoping reviews**. *Research Synthesis Methods* 2013:n/a-n/a.

Sun Y, Yang Y, Zhang H, Zhang W, Wang Q: **Towards evidence-based ontology for supporting Systematic Literature Review**. In: *Proceedings of the EASE Conference 2012: 2012 2012; Ciudad Real Spain*: IET; 2012.

Thomas J, O'Mara A: **How can we find relevant research more quickly?** In: *NCRM MethodsNews.* UK: NCRM; 2011: 3.

Tomassetti F, Rizzo G, Vetro A, Ardito L, Torchiano M, Morisio M: **Linked data approach for selection process automation in systematic reviews**. In: *Evaluation & Assessment in Software Engineering (EASE 2011), 15th Annual Conference on: 2011 2011; Durham*; 2011: 31-35.

Wallace B, Small K, Brodley C, Lau J, Schmid C, Bertram L, Lill C, Cohen J, Trikalinos T: **Toward modernizing the systematic review pipeline in genetics: efficient updating via data mining**. *Genetics in Medicine* 2012, **14**:663-669.

Wallace B, Small K, Brodley C, Lau J, Trikalinos T: **Modeling Annotation Time to Reduce Workload in Comparative Effectiveness Reviews**. In: *Proc ACM International Health Informatics Symposium: 2010 2010*; 2010: 28-35.

Wallace B, Small K, Brodley C, Lau J, Trikalinos T: **Deploying an interactive machine learning system in an evidence-based practice center: abstrackr**. In: *Proceedings of the 2nd ACM SIGHIT International Health Informatics Symposium: 2012 2012*: ACM; 2012: 819-824.

Wallace B, Small K, Brodley C, Trikalinos T: **Active Learning for Biomedical Citation Screening**. In: *KDD 2010: 2010 2010; Washington USA*; 2010.

Wallace B, Small K, Brodley C, Trikalinos T: **Who Should Label What? Instance Allocation in Multiple Expert Active Learning**. In: *Proc SIAM International Conference on Data Mining: 2011 2011*; 2011: 176-187.

Wallace B, Trikalinos T, Lau J, Brodley C, Schmid C: **Semi-automated screening of biomedical citations for systematic reviews**. *BMC Bioinformatics* 2010, **11**(55).

Yu W, Clyne M, Dolan S, Yesupriya A, Wulf A, Liu T, Khoury M, Gwinn M: **GAPscreener: an automatic tool for screening human genetic association literature in PubMed using the support vector machine technique**. *BMC Bioinformatics* 2008, **205**(9).

# Appendix D - Characteristics of included studies

| **Short Title** | **Number of reviews** | **Type of review** | **Study type** | **Compari-sons** | **Classifiers evaluated** | **Feature extraction approaches evaluated** | **Overall results/ conclusions (stated by authors)** |
| --- | --- | --- | --- | --- | --- | --- | --- |
| Bekhuis (2010) | 1 | • 'New' reviews | • Retro-spective simulation (used completed review) | • Classifiers/ algorithms • Training set size • Kernels | • EvoSVM • Other | • Bag-of-words | EvoSVM with a radial or Epanechnikov kernel may be an appropriate classifier when observational studies are eligible for inclusion in a systematic review |
| Bekhuis (2012) | 2 | • 'New' reviews | • Retro-spective simulation (used completed review) | • Classifiers/ algorithms • Feature extraction • Views (e.g., T&A, MeSH) | • SVM • EvoSVM • Naive Bayes • CNB • k-nearest neighbour | • Bag-of-words • N-grams • Other | In general, there appears to be a complex interaction between classiﬁer, citation portion, and feature set...  [Although] EvoSVM with a nonlinear kernel is promising, the runtimes are much longer than for cNB. In the near term, cNB may be the better choice to semi-automate citation screening, especially when the number of citations is large. |
| Bekhuis (2014) | 5 | • 'New' reviews • Updates | • Retro-spective simulation (used completed review) | • Feature extraction • Other opti-misations | • CNB | • LDA • Other | Although tests of ranked performance averaged over reviews suggested that the alphanumeric + set was best, post hoc pairwise comparisons indicated its statistical equivalence with the alphabetic set. |
| Choi (2012) | 145 | • 'New' reviews | • Retro-spective simulation (used completed review) | • Classifiers/ algorithms • Feature extraction • Kernels • Other opti-misations | • SVM • Naive Bayes | • Other | Compared to relevance or quality ranking alone, our re-ranking methodologies increased the performance impressively. [p. 87]  Results in Table 7 show that the Borda-fuse re-ranking algorithm had the highest macro-averaged precision [MAP]. |
| Cohen (2006) | 1, tested for four document triage tasks | • 'New' reviews | • Retro-spective simulation (used completed review) | • Classifiers/ algorithms • Number of features • Other opti-misations | • Other | • Bag-of-words | SVM by itself did not produce good results on these biomedical text classification tasks. However, the combination of chi-square binary feature selection, corrected cost-proportionate rejection sampling with a linear SVM, repeating the resampling process and combining the repetitions by voting is an approach that uniformly produces leading edge performance across all four tasks.  Feature set reduction using chi-square produced consistently better results than using all features. |
| Cohen et al. (2006) | 15 | • 'New' reviews | • Retro-spective simulation (used completed review) | • Classifiers/ algorithms | • SVM | • Other | A reduction in the number of articles needing manual review was found for 11 of the 15 drug review topics studied. For three of the topics, the reduction was 50% or greater. |
| Cohen (2008) | 15 | • Updates | • Retro-spective simulation (used completed review) | • Feature extraction • Views (e.g., T&A, MeSH) | • SVM | • N-grams • Other | The best feature set used a combination of n-gram and MeSH features. NLP-based features were not found to improve performance. Furthermore, topic-specific training data usually provides a significant performance gain over more general SR training.  Since extracting UMLS CUI features with MMTx is a computationally and time-intensive operation, and extracting n-grams is fast and simple, n-gram based features, in combination with MeSH terms, are to be preferred. Also, while inclusion of n-gram features was helpful in achieving maximum performance, there was no increased benefit in going from 2-gram to 3- or 4-gram length features. |
| Cohen (2009) | 24 | • Updates | • Retro-spective simulation (used completed review) | • Training set size • Topic specific vs general training data | • SVM | • N-grams | Overall, the hybrid system significantly outperforms the baseline system when topic-specific training data are sparse. Using 1/128 th of the available topic-specific data for training resulted in improved performance for 23 of the 24 topics.  The hybrid system will either improve performance, sometimes greatly, or not make much difference. |
| Cohen (2010) | 18 | • Updates | • Prospec-tive | • No comparison | • SVM | • N-grams | In general, the AUC measures are high, well over 0.80.  Sometimes, because of training set sizes, the performance can actually be better than predicted. For topics with significant changes in focus or breadth, performance may suffer. |
| Cohen (2011) | Linked study | Linked study | • Retro-spective simulation (used completed review) | • Classifiers/ algorithms | • SVM  • VP  • FCNB/WE | • Bag-of-words | The SVM outperformed for 12/15 reviews.  …the SVM approach is inferior to our prior VP results for the attention deﬁcit hyperactivity disorder (ADHD) topic, and that FCNB/WE is superior to both SVM and VP for the opioids topic, especially given that the SVM AUC measure is about 0.90 for both of these topics. Both the ADHD and opioids topics have very low article inclusion rates (2.4% and 0.8% respectively) and a relatively small number of positive samples (20 and 15 respectively). |
| Cohen (2012) | 9 | • Updates | • Prospec-tive | • Classifiers/ algorithms • Other opti-misations | • SVM | • N-grams | While we were able to consistently achieve the target recall of 0.55 on the training sets, recall performance varied widely on the test sets, from a low of 0.134 on AtypicalAntipsycho- tics to a high of 1.0 on NasalCorticosteroids . Precision also varied greatly, both on the training data as well as the test set, varying from a low of 0.306 on the Nasal- Corticosteroids test collection to a high of 0.800 on ProtonPumpInhibitors  While the number of update-motivating publications annotated for each topic varies quite a bit, the overall rate of alerts that need to be monitored is small, with most of the motivating publications recognized and lead ing to a correct alert. |
| Dalal (2013) | 2 | • Updates | • Retro-spective simulation (used completed review) | • Classifiers/ algorithms | • Other | • Unclear/ Not stated | GLMnet performed slightly better than GBM in this context, but overall model performance was similar despite their substantial theoretical differences.  We achieved good performance on both updates using statistical models that were empirically derived from earlier review inclusion judgments as well as explanatory variables selected using domain knowledge. |
| Felizardo (2011) | 1 | • 'New' reviews | • Prospec-tive  • Controlled trial (two human groups) | • No comparison | • VTM | • Other | Our results show that incorporating VTM in the SLR study selection activity reduced the time spent in this activity and also increased the number of studies correctly included. |
| Felizardo (2012) | 1 | • 'New' reviews | • Prospec-tive  • Controlled trial (two human groups) | • No comparison | • VTM | • Bag-of-words | The VTM sped up the process of selecting studies, but accuracy was the same as manual screening |
| Felizardo (2012) | 1 | Linked study | Linked study | Linked study | • VTM | Linked study | Authors report a statistically significant difference between groups in terms of performance (time taken to screen) but not effectiveness (number of primary studies correctly/incorrectly included/excluded). Also concluded that "the level of experience in researching can help to improve the effectiveness" (p. 99). This is because PhD students tended to have better performance than the Masters students |
| Felizardo (2013) | 1 | • 'New' reviews | • Prospec-tive  • Controlled trial (two human groups) | • No comparison | • VTM | • Other | From p. 177 of thesis version of document: "... the VTM approach can lend useful support to the primary study selection and selection review activities of SLRs". |
| Fiszman (2008) | 1, although tested on 4 questions (items were classified as relevant to each question) | • 'New' reviews | • Retro-spective simulation (used completed review) | • No comparison | • Semantic model | • Other | [Fiszman 2008.pdf] Page 1: The overall performance of the system was 40% recall, 88% precision (F 0.5 -score 0.71), and 98% specificity. We show that relevant and nonrelevant citations have clinically different semantic characteristics and suggest that this method has the potential to improve the efficiency of the literature review process in guideline development. |
| Fiszman (2010) | 1 | • 'New' reviews | • Prospec-tive | • Views (e.g., T&A, MeSH) | • Semantic model | • Other | [Fiszman 2010.pdf] Page 1: the overall performance of the system was 56% recall, 91% precision (F 0.5 -score 0.81). If quality of the evidence is not taken into account, performance drops to 62% recall, 79% precision (F 0.5 -score 0.75). |
| Frunza (2010) | 1 | • 'New' reviews | • Prospec-tive | • Classifiers/ algorithms • Feature extraction • Topic specific vs general training data | • CNB • Other | • Bag-of-words • Other | The global method achieves good results in terms of precision while the best recall is obtained by the per-question method. |
| Frunza (2011) | 1 | • 'New' reviews | • Retro-spective simulation (used completed review) | • Feature extraction • Topic specific vs general training data | • CNB | • Bag-of-words • Other | Overall, the best results were obtained by using the per-question method with the 2-vote scheme, including BOW representation with or without UMLS features. The results obtained by the three-vote scheme UMLS representation are close to the results obtained by the two-vote scheme, but F-measure results indicate that the 2-vote scheme is superior. Other per- question settings obtained better levels of recall... but the level of precision is too low. |
| García (2014) | 15 | • 'New' reviews | • Retro-spective simulation (used completed review) | • Classifiers/ algorithms • Number of features • Feature extraction • Views (e.g., T&A, MeSH) | • SVM • Naive Bayes • k-nearest neighbour • Other | • Other | Results are generally positive in terms of overall precision and recall measurements, reaching values of up to 84%. It is also revealing in terms of how using only article titles provides virtually as good results as when adding article abstracts.  From p. 1506: "In general, SVM clearly showed superiority over the rest of classifiers, not only in classification performance but in the number of required features to perform well."  From p. 1507: "Naive bayes offered the lowest rate of mistakes in the form of FN for any type of article, whereas SVM performed as well when using only titles then appending abstracts". |
| Jonnalagadda (2013) | 34 | • 'New' reviews | • Prospec-tive | • Classifiers/ algorithms | • Semantic model | • Vector Space | Across the 15 topics we examined, our system was not able to assure a high rate of recall (90%–95%) with a substantial reduction (40%) in workload reliably. |
| Kim (2012) | 1 | • 'New' reviews | • Retro-spective simulation (used completed review) | • Views (e.g., T&A, MeSH) • Topic specific vs general training data | • SVM | • Other | MeSH + publication type combination was concluded as the best performing feature content  [Kim 2012.pdf] Page 1: The system using the combination of included and commonly excluded articles performed better than the combination of included and excluded articles in all of the procedure topics. |
| Kouznetsov (2009) | 1 | • 'New' reviews | • Retro-spective simulation (used completed review) | • Classifiers/ algorithms • Feature extraction • Other opti-misations | • Naive Bayes • CNB • Regress-ion based • Other | • Bag-of-words • SOSCO | This shows that our method achieves a significant workload reduction, while maintaining the required performance level.  we achieved a much better performance when we use an ensemble (committee) of algorithms.  Complement Naïve Bayes outperformed the Voting Perceptron results reported by [9] on 12 of the 15 datasets. |
| Kouznetsov (2010) | 1 | • 'New' reviews | • Prospec-tive | • Feature extraction • Other opti-misations | • Naive Bayes • CNB • Regress-ion based • Other | • Bag-of-words • SOSCO | the classifier committee formed by applying the projection method of classifiers evaluation significantly over performed the validation committees that consist of the same number of algorithms arbitrary included from the same list of pre-selected classifiers. |
| Ma (2007) | 1 | • 'New' reviews | • Retro-spective simulation (used completed review) | • Classifiers/ algorithms • Feature extraction • Other opti-misations | • SVM • Naive Bayes • CNB • Other | • Bag-of-words | By using an active learning technique, we saved 86% of the effort required to label the training examples. The best testing result was obtained by combining the feature selection method Modified BNS, the sample selection method clustering-based sample selection and active learning with the Naive Bayes as classifier. |
| Malheiros (2007) | 1 | • 'New' reviews | • Prospec-tive  • Controlled trial (two human groups) | • No comparison | • VTM | • Unclear/ Not stated | p. 253: "...VTM could make the systematic review process more effective... The use of visualization allowed for more information to be processed at once.” |
| Martinez (2008) | 17 | • 'New' reviews | • Retro-spective simulation (used completed review) | • No comparison | • Regress-ion based | • Bag-of-words | we explored the use of ranked queries and text classification for better retrieval of the relevant documents. We found that different keyword-search strategies can reach recall that is comparable and sometimes better than the costly boolean queries. |
| Matwin (2010) | 15 | • 'New' reviews | • Retro-spective simulation (used completed review) | • Classifiers/ algorithms | • CNB • Other | Bag-of-words | We have shown how to modify CNB to emphasize the high recall on the minority class, which is a requirement in classi ﬁ cation of systematic reviews. The result, which we have called FCNB, is able to meet the restrictive requirement level of 95% recall that must be achieved. At the same time, we found that FCNB leads to better results in reducing the workload of systematic review preparation than the results previously achieved with the VP method. Moreover, FCNB can achieve even better performance results when machine-performed WE is applied. FCNB provides better interpretability than the VP approach, 1 and is far more efficient than the SVM classifier |
| Matwin (2011) | 15 | Linked study | • Retro-spective simulation (used completed review) | • Classifiers/ algorithms | • SVM • Naive Bayes | Linked study | we want to comment brie ﬂ y on the performance of FCNB/weight engineering (WE) on the Opioids dataset. As this" "dataset has a very high imbalance (very low inclusion rate), it is encouraging to see that the FCNB/WE method, which, as we discuss in our paper, has been engineered specifically to work well with such imbalanced data, indeed performs better than the standard SVM. |
| Matwin (2012) | 13 | • 'New' reviews | • Retro-spective simulation (used completed review) | • Classifiers/ algorithms | • SVM • Naive Bayes | • Unclear/ Not stated | using MNB as opposed to SVM appears to be appreciably faster without a significant loss in performance. |
| Miwa (2014) | 7 (3 medicine, 4 social science) | • 'New' reviews | • Retro-spective simulation (used completed review) | • Other opti-misations | • SVM | • Bag-of-words • LDA | The results show that the certainty criterion is useful for finding relevant documents, and weighting positive instances is promising to overcome the data imbalance problem in both data sets. Latent dirichlet allocation (LDA) is also shown to be promising when little manually-assigned information is available. |
| Razavi (2009) | 1 | • 'New' reviews | • Retro-spective simulation (used completed review) | • No comparison | • Other | • Bag-of-words • SOSCO | Since the machine learning prediction performance is generally on the same level as the human prediction performance, using the described system will lead to significant workload reduction for the human experts involved in the systematic review process. |
| Shemilt (2013) | 2 | • 'New' reviews | • Prospec-tive  • 'Case study' | • No comparison | • SVM • Other | • Unclear/ Not stated | reduced manual screening workload by 90% (CA) and 88% (EE) compared with conventional screening (absolute reductions of ≈ 430 000 (CA) and ≈ 378 000 (EE) records). |
| Sun (2012) | 1 | • 'New' reviews | • Prospec-tive  • 'Case study' | • No comparison | • Other | • Other | 11 papers are identified at last. Manual selection is rather time consuming. The total time used is 35 Person Hours . Using COSONT, we select the same 11 studies but time used by COSONT could be ignored. |
| Thomas (2011) | 1 | • 'New' reviews | • Prospec-tive  • 'Case study' | • No comparison | • Other | • Unclear/ Not stated | this method has enabled us to identify the expected number of relevant studies with only 25% of the usual manual work |
| Tomassetti (2011) | 1 | • 'New' reviews | • Retro-spective simulation (used completed review) | • No comparison | • Naive Bayes | • Bag-of-words | the process presented in this paper could reduce the work load of 20% with respect to the work load needed in the fully manually selection, with a recall of 100%. |
| Wallace (2010) | 1 | • 'New' reviews | • Prospec-tive | • Other opti-misations | • SVM | • N-grams | we demonstrated that normalizing these scores by the predicted time it will take to label the corresponding document results in a better performing system. Moreover, we presented a simple spline regression that incorporates document length and the order in which a document is labeled as predictive variables. The spline serves as a simple model for the annotator's learning rate. The coeffcients for this model can be learned online, as AL is ongoing. We showed that using this `return on investment' approach results in better performance in the same amount of time, compared with the greedy strategy. |
| Wallace (2010a) | 3 | • 'New' reviews | • Retro-spective simulation (used completed review) | • Other opti-misations | • SVM | • Bag-of-words | our algorithm is able to reduce the number of citations that must be screened manually by nearly half in two of these, and by around 40% in the third, without excluding any of the citations eligible for the systematic review. |
| Wallace (2010b) | 3 (but not all experiments conducted on all datasets) | • 'New' reviews | • Retro-spective simulation (used completed review) | • Views (e.g., T&A, MeSH) • Other opti-misations | • SVM | • Bag-of-words • Additional reviewer specified terms | Our findings suggest that the expert can, and should, provide more information than instance labels alone. |
| Wallace (2011) | 2 | • 'New' reviews | • Prospec-tive | • Other opti-misations | • SVM | • Unclear/ Not stated | Our meta-cognitive strategy out- performed strong baselines, including a previously pro- posed approach to MEAL, on both sentiment analysis and biomedical citation screening tasks. |
| Wallace (2012a) | 4 | • Updates | • Retro-spective simulation (used completed review) | • No comparison | • SVM | • Bag-of-words | The semi-automated system reduced the number of citations that would have needed to be screened by a human expert by 70–90%, a substantial reduction in workload, without sacrificing comprehensiveness. |
| Wallace (2012b) | 2 | • 'New' reviews | • Prospec-tive  • 'Case study' | • No comparison | • SVM | • Unclear/ Not stated | on both reviews for which the classification component of the abstrackr system has been deployed, it reduced workload (the number of citations that needed to be manually screened) by about 40% without wrongly excluding any relevant reviews, i.e., the sensitivity of the classifier was 100%. |
| Yu (2008) | 1 | • 'New' reviews • Updates | • Retro-spective simulation (used completed review) | • Other opti-misations | • SVM | • Other | Weighted SVM feature selection based on a keyword list obtained by the two- way z score method demonstrated the best screening performance, achieving 97.5% recall, 98.3% specificity and 31.9% precision in performance testing. Compared with the traditional screening process based on a complex PubMed query, the SVM tool reduced by about 90% the number of abstracts requiring individual review by the database curator. The tool also ascertained 47 articles that were missed by the traditional literature screening process during the 4-week test period. |
