# Supplementary material for: Using text mining for study identification in systematic reviews: a systematic review of current approaches
Source: Syst Rev. 2015 Jan 14;4(1):5. doi: 10.1186/2046-4053-4-5 (PMC4320539; doi:10.1186/2046-4053-4-5)
Supplement: Supplementary file 2 — Additional file 2: Flow diagram. (DOC 58 KB) [file 13643_2014_321_MOESM2_ESM.doc]

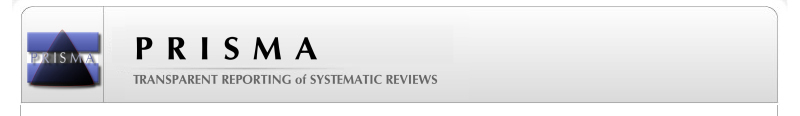
**PRISMA Flow Diagram**

**Screening**

**Included**

**Eligibility**

**Identification**

Records identified through database searching
(n = 1,434 )

Additional records identified through other sources
(n = 2 )

Records after duplicates removed
(n = 1,253 )

Records screened
(n = 1,253 )

Records excluded
(n = 1,184 )

Full-text articles assessed for eligibility
(n = 69 )

Full-text articles excluded
(n = 25 )

Not about text mining methods or metrics (2)

Not relevant to systematic reviews & screening (11)

General discussion (not evaluation) of the use of text mining for screening (12)

Studies included in qualitative synthesis
(n = 44 )

Studies included in quantitative synthesis (meta-analysis)
(n = 0 )
